# Design and deployment of the STEEER-AF trial to evaluate and improve guideline adherence: a cluster-randomized trial by the European Society of Cardiology and European Heart Rhythm Association

**DOI:** 10.1093/europace/euae178

**Published:** 2024-06-28

**Authors:** Maciej Sterliński, Karina V Bunting, Giuseppe Boriani, Serge Boveda, Eduard Guasch, Lluís Mont, Kim Rajappan, Philipp Sommer, Samir Mehta, Yongzhong Sun, Chris P Gale, Colinda van Deutekom, Isabelle C Van Gelder, Dipak Kotecha, STEEER-AF Trial Team, Yann Allali, Asgher Champsi, Thomas Deneke, Kaitlyn Greeley, Benoît Guy-Moyat, Mikael Laredo, Alastair Mobley, Maximina Ventura, Mary Stanbury, Trudie Lobban, Thompson Robinson, Tatjana Potpara, Eloi Marijon, Pascal Defaye, Pierre Baudinaud, Simon Kochhaeuser, Ursula Rauch, Moritz F Sinner, Marco Proietti, Igor Diemberger, Vincenzo Russo, Stanislaw Tubek, Piotr Buchta, Pawel Balsam, Eusebio García-Izquierdo, Ivo Roca Luque, Jose M Guerra, Dewi Thomas, Afzal Sohaib, Mark J Davies, Olivier Piot, William Escande, Christian De Chillou, Maxime De Guillebon, Frédéric Anselme, Andrea Cianci, Rodrigue Garcia, Philippe Maury, Dominique Pavin, Estelle Gandjbakhch, Frédéric Sacher, Karim Hasni, Fabien Garnier, Charles Guenancia, Nicolas Lellouche, Stephan Willems, Martin Borlich, Andreas Metzner, Hans-Holger Ebert, Dong-In Shin, David Duncker, Stefan G Spitzer, Peter Nordbeck, Roland R Tilz, Andrea Mazza, Cinzia Valzania, Margherita Padeletti, Matteo Bertini, Giuseppe Boriani, Jacopo F Imberti, Stefano Fumagalli, Antonio Rapacciuolo, Monika Lica Gorzynska, Adam Gorlo, Marcin Kostkiewicz, Grzegorz Sobieszek, Andrzej S Skrzyński, Robert Gajda, Hanna Wilk-Manowiec, Jaroslaw Blicharz, Wiktor K Gmiński, Tomasz Czerski, Felipe Bisbal, Ignasi Anguera, Teresa Lozano, Joaquin Osca, Jose L Merino, Naiara Calvo, Juan Fernández-Armenta, Juan Acosta, Nuria Rivas-Gandara, Pilar Cabanas, Emilce Trucco, Richard Bond, Richard Ang, Shawn A A Morais, Fu Siong Ng, Mattew G D Bates, Michala Pedersen, Daniel T Raine, Manish Kalla, Matthew J Lovell, Malcolm Finlay, Arif Hasan Bhuiyan, Norman Qureshi, Hein Heidbuchel, Wolfram Döhner, Bernard Iung, Susanna Price, Helmut Pürerfellner, Barbara Casadei, Paulus Kirchhof, Alex R Lyon, Winston Banya, Robert Hatala, Pekka Raatikainen

**Affiliations:** Arrhythmia Center, National Institute of Cardiology, Warsaw, Poland; Institute of Cardiovascular Sciences, University of Birmingham, Medical School, Vincent Drive, Birmingham B15 2TT, UK; Cardiology Department, University Hospitals Birmingham NHS Foundation Trust, Birmingham, UK; Division of Cardiology, Department of Biomedical, Metabolic and Neural Sciences, Policlinico di Modena, University of Modena and Reggio Emilia, Modena, Italy; Heart Rhythm Management Department, Cardiology, Clinique Pasteur, Toulouse, France; Cardiology Department, Clinic Barcelona, Barcelona, Spain; Institut d'Investigacions Biomèdiques August Pi i Sunyer (IDIBAPS), Barcelona, Spain; Medicine Department, Universitat de Barcelona, Barcelona, Spain; Centro de Investigación Médica en Red—Enfermedades Cardiovasculares (CIBERCV), Madrid, Spain; Institut d'Investigacions Biomèdiques August Pi i Sunyer (IDIBAPS), Barcelona, Spain; Centro de Investigación Médica en Red—Enfermedades Cardiovasculares (CIBERCV), Madrid, Spain; Arrhythmias Section, Cardiovascular Clinical Institute, Hospital Clínic, Universitat de Barcelona, Barcelona, Spain; Cardiology Department, Oxford University Hospitals NHS Foundation Trust, Headley Way, Oxford, UK; Clinic for Electrophysiology, Heart and Diabetes Center NRW, Georgstraße 11, Bad Oeynhausen, Germany; Birmingham Clinical Trials Unit (BCTU), Institute of Applied Health Research, Birmingham, UK; Birmingham Clinical Trials Unit (BCTU), Institute of Applied Health Research, Birmingham, UK; Leeds Institute for Cardiovascular and Metabolic Medicine, University of Leeds, Leeds, UK; Leeds Institute of Data Analytics, University of Leeds, Leeds, UK; Department of Cardiology, Leeds Teaching Hospitals, Leeds, UK; Department of Cardiology, University of Groningen, University Medical Centre Groningen, Groningen, The Netherlands; Department of Cardiology, University of Groningen, University Medical Centre Groningen, Groningen, The Netherlands; Institute of Cardiovascular Sciences, University of Birmingham, Medical School, Vincent Drive, Birmingham B15 2TT, UK; Cardiology Department, University Hospitals Birmingham NHS Foundation Trust, Birmingham, UK; NIHR Birmingham Biomedical Research Centre, Heritage Building, Mindelsohn Way, Birmingham B15 2TH, UK; West Midlands NHS Secure Data Environment, University Hospitals Birmingham NHS Foundation Trust, Mindelsohn Way, Birmingham B15 2GW, UK

**Keywords:** Atrial fibrillation, Guideline adherence, Stroke prevention, Rhythm control, Trial

## Abstract

**Aims:**

The aim is to describe the rationale, design, delivery, and baseline characteristics of the Stroke prevention and rhythm control Treatment: Evaluation of an Educational programme of the European society of cardiology in a cluster-Randomized trial in patients with Atrial Fibrillation (STEEER-AF) trial.

**Methods and results:**

STEEER-AF is a pragmatic trial designed to objectively and robustly determine whether guidelines are adhered to in routine practice and evaluate a targeted educational programme for healthcare professionals. Seventy centres were randomized in six countries (France, Germany, Italy, Poland, Spain, and UK; 2022–23). The STEEER-AF centres recruited 1732 patients with a diagnosis of atrial fibrillation (AF), with a mean age of 68.9 years (SD 11.7), CHA_2_DS_2_-VASc score of 3.2 (SD 1.8), and 647 (37%) women. Eight hundred and forty-three patients (49%) were in AF at enrolment and 760 (44%) in sinus rhythm. Oral anticoagulant therapy was prescribed in 1543 patients (89%), with the majority receiving direct oral anticoagulants (1378; 89%). Previous cardioversion, antiarrhythmic drug therapy, or ablation was recorded in 836 patients (48.3%). Five hundred fifty-one patients (31.8%) were currently receiving an antiarrhythmic drug, and 446 (25.8%) were scheduled to receive a future cardioversion or ablation. The educational programme engaged 195 healthcare professionals across centres randomized to the intervention group, consisting of bespoke interactive online learning and reinforcement activities, supported by national expert trainers.

**Conclusion:**

The STEEER-AF trial was successfully deployed across six European countries to investigate guideline adherence in real-world practice and evaluate if a structured educational programme for healthcare professionals can improve patient-level care.

**Clinical Trial Registration:**

Clinicaltrials.gov, NCT04396418.

## Graphical Abstract

**Graphical Abstract:**
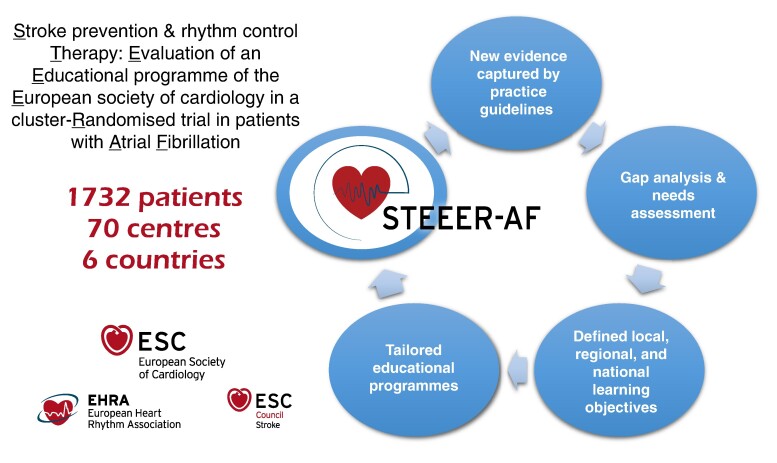
The STEEER-AF trial in the context of the European Society of Cardiology roadmap for cardiovascular education (https://doi.org/10.1093/eurheartj/ehy058).^11^

**What’s new?**

- The Stroke prevention and rhythm control Treatment: Evaluation of an Educational programme of the European society of cardiology in a cluster-Randomized trial in patients with Atrial Fibrillation (STEEER-AF) trial is a cluster-randomized trial designed to evaluate guideline adherence and test an educational programme for healthcare professionals treating patients with atrial fibrillation (AF).
- The trial has successfully recruited 1732 patients with AF from 70 centres across the 6 European countries.
- This paper describes the design and deployment of the STEEER-AF trial, the educational intervention, and the process for determining patient-level adherence to guidelines on stroke prevention and rhythm control.

## Introduction

Atrial fibrillation (AF) is one of the most prevalent cardiovascular conditions and set to double further over the next few decades.^1,2^ Clinical practice guidelines, such as those by the European Society of Cardiology (ESC), can assist healthcare professionals to apply optimal care to patients.^3,4^ However, guidelines are often poorly implemented, with education and training of healthcare staff identified as major barriers.^5,6^ In particular, the management of stroke prevention and rhythm control for patients with AF are complex and rely on the education and knowledge base of a broad range of healthcare professionals working as a team.^7^ Guideline-adherent treatment has been shown to improve the outcomes of patients with AF, including lower rates of mortality, incident stroke, and major bleeding,^8–10^ reinforcing the importance of guideline implementation.

The Stroke prevention and rhythm control Treatment: Evaluation of an Educational programme of the European society of cardiology in a cluster-Randomized trial in patients with Atrial Fibrillation (STEEER-AF) trial is a joint effort by the ESC, the European Heart Rhythm Association (EHRA) and the ESC Council on Stroke (*Graphical Abstract*). The primary objective of the trial is to test whether a structured educational programme for healthcare professionals can improve guideline-adherent provision of care for patients with AF. This report details the rationale, design, and delivery of the STEEER-AF trial and the characteristics of the participants enrolled across the six European countries.

## STEEER-AF rationale and design

The true value of educational interventions for healthcare professionals are poorly understood, with surveys and other observational assessments providing very limited evaluation. Considering the time, effort, and expense required to train a clinical workforce and keep these staff updated,^11^ a formal evaluation of the effectiveness of education that targets AF care was needed. The STEEER-AF trial was therefore designed as a pragmatic, parallel group, two-arm, international, cluster-randomized controlled trial (RCT), the first RCT sponsored by the ESC. The primary objective was to determine whether a comprehensive and structured educational programme for healthcare professionals treating patients with AF, compared with no added education, would improve guideline-adherent provision of patient-level treatments relating to stroke prevention and rhythm control.

The STEEER-AF trial was initially designed to evaluate a blended mode educational intervention (online and face to face) using the 2016 ESC Guidelines on the management of AF.^4^ The programme was developed jointly by the ESC Education Committee, EHRA, and the ESC Council on Stroke. Due to the coronavirus pandemic, the start of the trial was delayed, the intervention reformatted into an online-only approach supported by expert national trainers, and the educational content and evaluation redesigned to incorporate changes in the 2020 ESC Guidelines.^3^

The STEEER-AF trial is embedded and led within six large countries representing the ESC—France, Germany, Italy, Poland, Spain, and the UK. National coordinators for each country were proposed by EHRA, who were tasked with engaging a broad range of centres that treat patients with AF and selecting a local principal investigator (PI) for each site. Each PI then nominated healthcare professionals in their institution (investigators), who were a range of staff across different specialities, with no more than a third of investigators seeing patients with AF on a daily basis. These investigators then recruited up to 25 patients per centre who had a diagnosis of AF, were able to provide informed consent, and did not have exclusion criteria (pregnant, planning to be pregnant at the time of consent, participating in another clinical trial, or life expectancy < 2 years). The trial is supported centrally by ESC staff, a Trial Management Group, and a Trial Steering Committee. Oversight was provided by a Data Monitoring Committee and Strategic Oversight Committee (see Supplementary material online, *Table S1*, for full details of the STEEER-AF team). The trial is being conducted in accordance with the guidelines for Good Clinical Practice and the Declaration of Helsinki, has regulatory approval from the Research Ethics Committees across Europe (see Supplementary material online, *Table S2*), and is registered at Clinicaltrials.gov (NCT04396418). The full protocol is provided in the Supplementary material online, *File*. A patient and public involvement team assisted in designing this trial to meet the needs of patients with AF and helped to write all patient-facing material.

### Cluster-randomized approach

**Figure 1:**
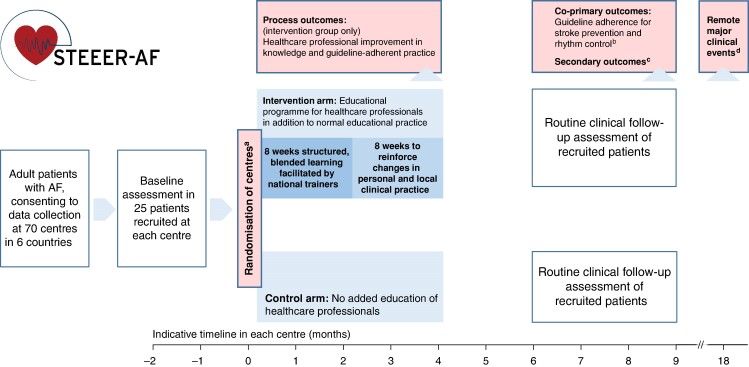
STEEER-AF trial flowchart. Process and outcomes in the STEEER-AF trial. ^a^Minimization algorithm accounting for country and guideline adherence for stroke prevention and rhythm control at baseline. ^b^Class I and III recommendations on stroke prevention and rhythm control therapy (ESC Guidelines on the management of AF). ^c^Proportion of relevant guidelines attained; appropriate use of anticoagulation; integrated AF approach; patient quality of life. ^d^Composite of mortality, stroke, TIA, pulmonary or systolic embolus, acute coronary syndrome/myocardial infarction, heart failure, and major or clinically relevant bleeding plus hospital admissions. AF, atrial fibrillation; ESC, European Society of Cardiology; STEEER-AF, Stroke prevention and rhythm control Treatment: Evaluation of an Educational programme of the European society of cardiology in a cluster-Randomized trial in patients with Atrial Fibrillation.

The STEEER-AF trial is a cluster-RCT, where centres rather than individuals are randomized to either the intervention group (structured education for healthcare professionals) or the control group (usual approaches to education of healthcare staff; *Figure 1*). A cluster design was needed to avoid contamination effects from the educational programme. Randomization was performed with a 1:1 ratio to intervention or control. A minimization algorithm was used to ensure balance by (i) country; (ii) cluster-specific mean for Class I and III guideline adherence to stroke prevention at baseline (<70 and ≥70%); and (iii) cluster-specific mean for Class I and III guideline adherence to rhythm control at baseline (<50 and ≥50%).

### Outcomes

The co-primary outcomes of the STEEER-AF trial are guideline adherence for stroke prevention therapy and rhythm control therapy, based on Class I and III ESC recommendations, and evaluated at 6–9 months after randomization as part of routine clinical care by the same site investigators. Secondary outcomes include the proportion of guidelines adhered to, the proportion of patients with appropriate anticoagulation, and patient-reported outcomes for integrated AF management and quality of life. Further (remote) follow-up is planned beyond 18 months after randomization to collect information on death and major adverse cardiovascular events. The full list of outcomes is presented in Supplementary material online, *Table S3*.

### Approaches to minimize bias

The randomized allocation was performed by the trial statistician blinded to the identity of the centres. Due to the nature of the intervention, it was not possible to blind hospital or health centre staff to the randomized allocation. However, to minimize the selection and ascertainment biases, centres were not randomized until they had recruited the required number of patients and completed the baseline electronic case report form (eCRF) managed by an independent contract research organization (Soladis, France). The eCRF was completed by the PI of that centre who was not involved in the care pathway for recruited participants in order to be an objective assessor of the clinical care received. Enrolled patients were asked to complete a short quality of life questionnaire (EQ-5D-5L) and to evaluate the integrated care they received (covering education, lifestyle and risk factors, treatment adherence, self-management, shared decision making, decision support tools, and multidisciplinary approaches). Guideline adherence was not disclosed to the PI or site staff at any point; instead, the completed eCRF data at baseline and 6–9 months follow-up were analysed by an algorithm to determine adherence to Class I and III ESC recommendations. The algorithms for defining guideline adherence for stroke prevention are presented in Supplementary material online, *Figures S2* and *S3*, and for rhythm control in Supplementary material online, *Figure S4*. The coordinating staff (chief investigators and Trial Steering Committee) were blinded to the randomized allocation of centres during the entire trial to minimize reporting and performance biases.

### Sample size calculations

Based on published observational surveys, 80% of the control patients were expected to receive guideline-adherent care for stroke prevention.^12,13^ A relative increase of 10% (i.e. absolute increase of 8% from 80 to 88%) was considered a clinically relevant improvement in guideline adherence for stroke prevention. The STEEER-AF trial was designed to have a power of 85% for this outcome, with sample size calculations based on an intracluster correlation coefficient of 0.04,^14,15^ two-sided alpha of 0.05, cluster size of 25 patients, 70 clusters with a total of 1750 patients, 10% loss to patient follow-up, and limited variation in cluster size (coefficient of variation of cluster size of 0.20). For the rhythm control outcome, estimates of the control group rate were 50%.^12,16^ With the same assumptions as above, the sample size would provide 85% power to detect an absolute increase in guideline adherence for rhythm control from 50 to 61%.

## STEEER-AF recruitment

**Figure 2:**
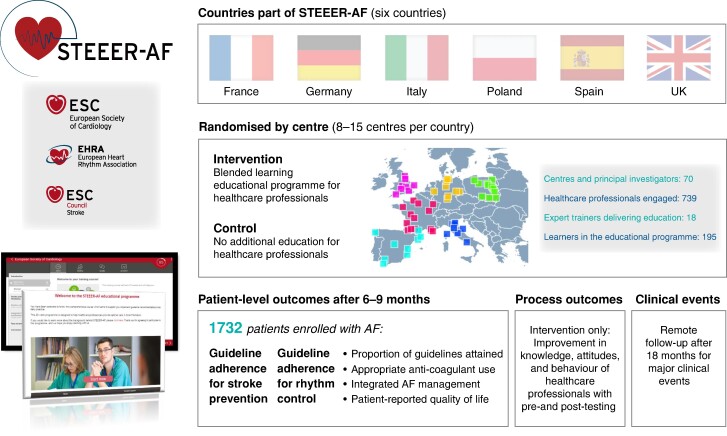
STEEER-AF deployment. Centres, patients, and healthcare professionals engaging with STEEER-AF across six countries. STEEER-AF, Stroke prevention and rhythm control Treatment: Evaluation of an Educational programme of the European society of cardiology in a cluster-Randomized trial in patients with Atrial Fibrillation.

Seventy centres across France, Germany, Italy, Poland, Spain, and the UK recruited patients with AF (*Figure 2*), with an average cluster size of 24.7 patients (coefficient of variation of cluster size of 0.06). Randomization of centres occurred between May 2022 and February 2023. In total, 739 healthcare professionals were engaged within the STEEER-AF programme and 1732 patients with AF were consented and enrolled. There were 18 expert trainers supervising 195 learners that participated in the educational intervention.

### Patient characteristics

The mean age of the 1732 trial participants was 68.9 years (SD 11.7), with 647 (37.4%) women. The type of AF was first diagnosed in 282 (16.3%), paroxysmal in 656 (37.9%), persistent in 484 (27.9%), long-standing persistent in 66 (3.8%), and permanent in 244 (14.1%). Hypertension requiring current treatment was present in 1207 patients (69.7%), a diagnosis of heart failure in 489 (28.2%), diabetes in 386 (22.3%), prior myocardial infarction in 166 (9.6%), and a history of stroke or transient ischaemic attack in 176 (10.2%). The mean CHA_2_DS_2_-VASc score was 3.2 (SD 1.8), and the CHA_2_DS_2_-VA score (excluding gender) was 2.8 (SD 1.7). At their enrolment visit, 843 patients (48.7%) were in AF and 760 (43.9%) in sinus rhythm. Other characteristics and measurements are presented in *Table 1* and medications and therapeutic approaches in *Table 2*.

**Table 1:** Baseline characteristics

Characteristic	*n* = 1732
**Patient recruitment by country (70 sites)**, *n (%)*	
France, 15 sites	375 (21.7%)
Germany, 11 sites	275 (15.9%)
Italy, 8 sites	200 (11.5%)
Poland, 12 sites	300 (17.3%)
Spain, 11 sites	275 (15.9%)
UK, 13 sites	307 (17.7%)
**Patient demographics**	
Age at enrolment, mean years (SD)	68.9 (11.7)
Women, *n* (%)	647 (37.4%)
**Type of AF**, *n (%)*	
First diagnosed	282 (16.3%)
Paroxysmal	656 (37.9%)
Persistent	484 (27.9%)
Long-standing persistent	66 (3.8%)
Permanent	244 (14.1%)
**Duration of AF**, *n (%)*	
≤1 year	682 (39.4%)
1–5 years	542 (31.3%)
>5 years	508 (29.3%)
**Rhythm on baseline ECG**, *n (%)*^a^	
Sinus rhythm	760 (43.9%)
AF	843 (48.7%)
**Comorbidities**, *n* (%)	
Hypertension requiring treatment	1207 (69.7%)
Diabetes mellitus	386 (22.3%)
History of stroke, TIA	176 (10.2%)
History of myocardial infarction	166 (9.6%)
Diagnosis of heart failure	489 (28.2%)
Diagnosis of COVID-19	272 (15.7%)
**Left ventricular systolic function**, *n (%)*	
Preserved (LVEF ≥50%)	1288 (74.4%)
Mildly reduced (LVEF 40–49%)	207 (12.0%)
Reduced (LVEF <40%)	237 (13.7%)
**Clinical measurements at baseline** ^b^	
Resting heart rate on ECG, beats/min (SD)	79.2 (23.2)
Systolic blood pressure, mmHg (SD)	131.8 (19.7)
Diastolic blood pressure, mmHg (SD)	77.8 (12.5)
Body mass index, kg/m^2^ (SD)	28.6 (5.8)
Creatinine, μmol/L (SD)	122.8 (243.4)

AF, atrial fibrillation; COVID-19, coronavirus disease 2019; ECG, electrocardiogram; LVEF, left ventricular ejection fraction; SD, standard deviation; TIA, transient ischaemic attack.

^a^129 patients had another rhythm or pacing at baseline.

^b^Missing data: 20 patients for ECG heart rate; 52 for systolic blood pressure; 53 for diastolic blood pressure; 38 for body mass index; 205 for creatinine.

**Table 2:** Baseline treatments

Characteristic, *n* (%)	*n* = 1732
Currently taking any antiplatelet drug	168 (9.7%)
Currently taking an oral anticoagulant	1543 (89.1%)
Direct oral anticoagulant	1378 (89.3%)
Vitamin K antagonist oral anticoagulant	165 (10.7%)
Absolute contraindication to anticoagulant therapy	24 (1.4%)
Left atrial appendage occlusion/excision	18 (1.0%)
Currently receiving any rate control medication	1364 (78.8%)
*Medication for rate control* ^a^	
Beta-blockers	1271 (73.4%)
Digoxin or digitoxin	115 (6.6%)
Diltiazem or verapamil	36 (2.1%)
Amiodarone	175 (10.1%)
History of pacemaker or device implantation	248 (14.3%)
History of atrioventricular node ablation	41 (2.4%)
Currently receiving antiarrhythmic drugs	551 (31.8%)
*Type of antiarrhythmic drug* ^a^	
Amiodarone	276 (15.9%)
Flecainide	143 (8.3%)
Propafenone	55 (3.2%)
Dronedarone	6 (0.3%)
Sotalol	26 (1.5%)
Other antiarrhythmic drug	62 (3.6%)
Previous catheter ablation	343 (41.0%)
Scheduled to receive cardioversion or ablation	446 (25.8%)

^a^Not mutually exclusive.

### Stroke prevention therapy at baseline

Oral anticoagulant therapy was prescribed in 1543 patients (89.1%). The majority were taking direct oral anticoagulants (DOAC; 1378 patients; 89.3%) rather than vitamin K antagonists (VKA; 165 patients; 10.7%). Antiplatelet therapy was prescribed in addition to an anticoagulant in 122 patients (7.0%). Very few individuals were noted as having an absolute contraindication to oral anticoagulant therapy (24 patients; 1.4%) or prior left atrial appendage occlusion (18 patients; 1.0%).

### Rhythm control approaches at baseline

Previous cardioversion, antiarrhythmic drug therapy, or ablation was recorded in 836 patients (48.3%). Five hundred fifty-one patients (31.8%) were currently receiving an antiarrhythmic drug (amiodarone and flecainide the most common), and 446 (25.8%) were scheduled to receive a cardioversion or ablation in the future. In total, 1156 patients (66.7%) had prior rhythm control, current use of antiarrhythmic drug therapy or were planned for future rhythm interventions.

## STEEER-AF intervention

The educational intervention is targeted towards stroke prevention, rhythm control, and integrated care, with learning modules translated for each participating country. Investigators in centres randomized to the intervention group form the learner cohort, supported in their use of the bespoke online platform by an expert trainer from that country. The intervention was developed by ESC Education, EHRA, external content leads, and the assistance of an independent medical education agency (Liberum IME, London, UK). The educational programme consists of direct and indirect approaches so that both the individual learner and their local colleagues can benefit from the educational intervention (see Supplementary material online, *Figure S1*):

1. Multiple-choice questions before the education (Week 1), providing a baseline assessment of Class I and III ESC recommendations pertaining to stroke prevention and rhythm control in AF
2. Online learning experience (Weeks 2–6), including interactive resources, case-based materials, videos on related topics, and additional reading lists
3. Multiple-choice questions after the education (Week 7), to ascertain change in knowledge and reinforce learning
4. Drafting a locally relevant ‘commitment to change plan (Week 7), to encourage a broad range of staff in each centre to manage patients with AF better
5. Remote small group workshops with the trainer (Week 8), with the aim of generating tangible action points
6. Reinforcing activities and iterative feedback on the ‘commitment to change plan (Weeks 9–15), including key messages (see Supplementary material online, *Figure S5*)
7. Survey of learners (Week 16), to provide feedback and value statements on the educational intervention

The education provided to healthcare staff at centres randomized to the intervention group is supplementary to any existing continued professional development. Centres randomized to the control group do not receive the educational intervention but are able to continue any existing programmes of professional development.

## Discussion

**Figure 3:**
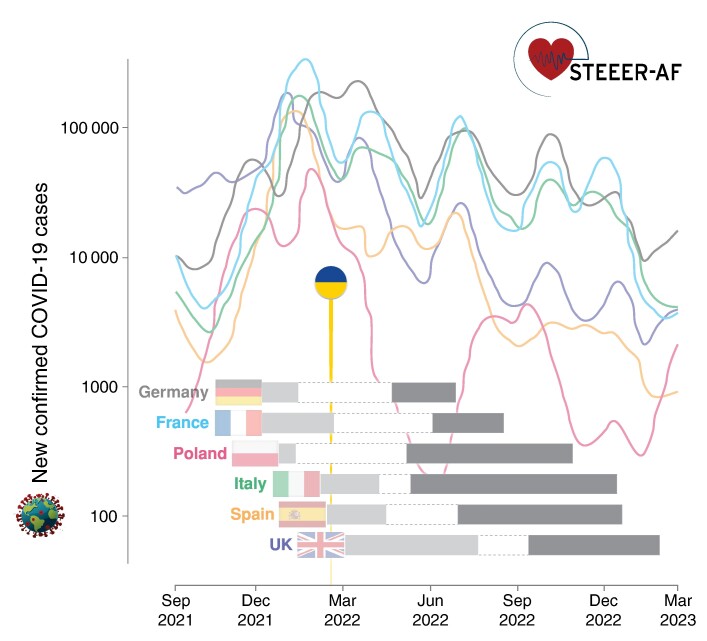
Global context during initiation of the STEEER-AF trial. Displayed for each country taking part in STEEER-AF are the activation of sites and start of patient recruitment (light grey bar) and randomization of centres after completion of patient recruitment (dark grey bars). Corresponding data for COVID-19 cases are displayed (7-day rolling average of confirmed cases from the World Health Organization COVID-19 dashboard; log scale) for Germany, France, Poland, Italy, Spain, and the UK. The round marker notes the start of refugee migration following the invasion of Ukraine. COVID-19, coronavirus disease 2019; STEEER-AF, Stroke prevention and rhythm control Treatment: Evaluation of an Educational programme of the European society of cardiology in a cluster-Randomized trial in patients with Atrial Fibrillation.

The STEEER-AF trial was designed to robustly evaluate and improve the implementation of clinical guidelines, a critical evidence gap in routine practice. The approach was supported and coordinated by the ESC and EHRA, not-for-profit organizations that represent clinicians, nurses, allied health professionals, and scientists working on behalf of the 57 national cardiac societies. Despite immense healthcare challenges such as the coronavirus pandemic and refugees from the Ukraine war, local STEEER-AF teams across France, Germany, Italy, Poland, Spain, and the UK have been able to successfully deliver a cluster-RCT (*Figure 3*), engaging with thousands of patients and healthcare professionals across Europe.

This paper outlines the key design features of the STEEER-AF trial that were essential to objectively evaluate the extent of guideline adherence in real-world clinical practice and test whether structured education could optimize patient-level guideline adherence. Atrial fibrillation was chosen as the topic for the ESC’s first clinical trial due to its association with preventable adverse outcomes,^17,18^ evolving options for management,^19–21^ and the observation of better prognosis when patient care is adherent to guidelines. The evidence base for guideline-adherent care is largely restricted to anticoagulation use in AF and previously based on non-randomized studies where selection biases were likely substantial.^22,23^ Subsequently, a cluster-RCT of 2281 patients across 5 countries used an educational programme to achieve higher rates of anticoagulant prescription and demonstrated a reduction in the secondary outcome of incident stoke [hazard ratio 0.48, 95% confidence interval (CI) 0.23–0.99; *P* = 0·04].^15^ The other principal reasons for investigating guideline adherence in AF were its increasing burden on society^24^ and that implementation of guidelines are sub-optimal in this condition with strategies for effective improvement in adherence known to be complex.^25^ Management is made more difficult in patients with AF due to the frequent and increasing occurrence of multiple comorbidities and frailty^24,26,27^ and the variable reasons for treatment initiation (symptom control vs. prognostic impact). Added to these issues are training and maintaining knowledge across a broad workforce to avoid barriers to guideline implementation.^5,6,28^

In the STEEER-AF trial, we focused on the main areas that have confounded guideline adherence, targeting stroke prevention and rhythm control, as well as how care can be integrated to provide maximal patient benefit. These were prioritized following a multinational mixed-methods study of 561 clinicians across the 6 STEEER-AF countries to understand educational needs that could affect the care of AF patients.^29^ Many of the physicians reported insufficient skills to use stroke risk assessment for management decisions, with considerable uncertainty in how to deal with anticoagulant therapy in complex patients and those with comorbidities. Whilst formal stroke risk scoring was commonly used by cardiologists (94%), it was substantially lower for neurologists (60%) and general practitioners (58%). Insufficient knowledge to select patients for AF ablation was disclosed by 61% of cardiologists, 98% of neurologists, and 87% of general practitioners. These issues have important clinical consequence, with a third of patients presenting with ischaemic stroke having known or newly detected AF,^30^ a higher risk of dementia due to subclinical cerebral damage,^31–33^ and AF leading to considerable symptom burden and poor quality of life for patients.^34^

The patients enrolled in the STEEER-AF trial were a good representation of usual clinical care for AF, which is often difficult to achieve in conventional RCTs. Age and comorbidity burden were similar to global registries of patients with AF, such as in 9816 patients from 831 centres across 26 countries^35^ and 8082 patients from 192 centres across 31 countries.^36^ The high rate of oral anticoagulant use, preference for DOACs rather than VKA, and low prevalence of absolute contraindications to anticoagulation were also consistent with large-scale observational cohorts.^37–39^ As expected from a trial recruiting patients admitted with AF, we saw more first-onset and paroxysmal AF than those with permanent AF. Data on the use of rhythm control are less easy to compare with other studies due to marked differences in patient populations and regional differences in who is able to receive (or be reimbursed) for rhythm control procedures. However, baseline declaration of rhythm control in the STEEER-AF population was broadly compatible with the 54% of patients receiving rhythm control in a sample of the EURObservational Research Programme registry from 250 centres across 27 countries.^40^

The STEEER-AF trial was a pragmatic randomized clinical trial, based within usual clinical care and therefore subject to variations in practice across and within countries. Although deploying an investigator-driven cluster-RCT across multiple countries was a challenge, the resulting findings will provide the necessary robustness to determine how healthcare professional education can impact on guideline adherence. The minimization algorithm was designed to avoid major imbalances in randomization by taking account of guideline adherence at baseline for the primary outcomes and across the different countries. Whereas design papers for clinical trials are typically published early in their deployment, this was not appropriate for the STEEER-AF trial as knowledge of the scoring process could have affected the study outcomes. With all primary outcome data at 6–9 months follow-up now collected, we are able to publish the schema and algorithms used to capture guideline adherence in the trial without adverse influence. The co-primary outcomes of the STEEER-AF trial will be presented at the ESC Congress in London on 01 September 2024.

## Conclusion

The STEEER-AF cluster-randomized trial was launched across the six European countries to objectively examine guideline adherence in routine clinical practice and test if a structured educational programme can improve adherence to guideline recommendations and the care of patients with AF.

## Supplementary Material

euae178_Supplementary_Data

## Data Availability

No additional data are available at this time.

## Appendix

**STEEER-AF Trial Team**

Includes supporting staff, patient and public representatives; education leads; principal investigators; oversight committees.

***Supporting staff:*** Yann Allali (France); Asgher Champsi (UK); Thomas Deneke (Germany); Kaitlyn Greeley (France); Benoît Guy-Moyat (France); Mikael Laredo (France); Alastair Mobley (UK); Maximina Ventura (UK).

***Patient and public representatives:*** Mary Stanbury (UK); Trudie Lobban (UK).

***Education leads:*** Tom De Potter (Belgium); Thompson Robinson (UK); Tatjana Potpara (Serbia); Eloi Marijon (France); Pascal Defaye (France); Pierre Baudinaud (France); Simon Kochhaeuser (Germany); Ursula Rauch (Germany); Moritz F. Sinner (Germany); Marco Proietti (Italy); Igor Diemberger (Italy); Vincenzo Russo (Italy); Stanislaw Tubek (Poland); Piotr Buchta (Poland); Pawel Balsam (Poland); Eusebio García-Izquierdo (Spain); Ivo Roca Luque (Spain); Jose M. Guerra (Spain); Dewi Thomas (UK); Afzal Sohaib (UK); Mark J. Davies (UK).

***Principal investigators:*** Olivier Piot (France); William Escande (France); Christian De Chillou (France); Maxime De Guillebon (France); Frédéric Anselme (France); Andrea Cianci (France); Rodrigue Garcia (France); Philippe Maury (France); Dominique Pavin (France); Estelle Gandjbakhch (France); Frédéric Sacher (France); Karim Hasni (France); Fabien Garnier (France); Charles Guenancia (France); Nicolas Lellouche (France); Stephan Willems (Germany); Martin Borlich (Germany); Andreas Metzner (Germany); Hans-Holger Ebert (Germany); Dong-In Shin (Germany); David Duncker (Germany); Stefan G. Spitzer (Germany); Peter Nordbeck (Germany); Roland R. Tilz (Germany); Andrea Mazza (Italy); Cinzia Valzania (Italy); Margherita Padeletti (Italy); Matteo Bertini (Italy); Jacopo F. Imberti (Italy); Stefano Fumagalli (Italy); Antonio Rapacciuolo (Italy); Monika Lica Gorzynska (Poland); Adam Gorlo (Poland); Marcin Kostkiewicz (Poland); Grzegorz Sobieszek (Poland); Andrzej S. Skrzyński (Poland); Robert Gajda (Poland); Hanna Wilk-Manowiec (Poland); Jaroslaw Blicharz (Poland); Wiktor K. Gmiński (Poland); Tomasz Czerski (Poland); Felipe Bisbal (Spain); Ignasi Anguera (Spain); Teresa Lozano (Spain); Joaquin Osca (Spain); Jose L. Merino (Spain); Naiara Calvo (Spain); Juan Fernández-Armenta (Spain); Juan Acosta (Spain); Nuria Rivas-Gandara (Spain); Pilar Cabanas (Spain); Emilce Trucco (Spain); Richard Bond (UK); Richard Ang (UK); Shawn A. A. Morais (UK); Fu Siong Ng (UK); Matthew G. D. Bates (UK); Michala Pedersen (UK); Daniel T. Raine (UK); Manish Kalla (UK); Matthew J. Lovell (UK); Malcolm Finlay (UK); Arif Hasan Bhuiyan (UK); Norman Qureshi (UK).

***Oversight committees:* Strategic oversight committee:** Hein Heidbuchel (Chair; Belgium); Wolfram Döhner (Germany); Bernard Iung (France); Susanna Price (UK); Helmut Pürerfellner (Austria); Barbara Casadei (UK); Paulus Kirchhof (Germany). **Data monitoring committee:** Alex R. Lyon (Chair; UK); Winston Banya (UK); Robert Hatala (The Slovak Republic); Pekka Raatikainen (Finland).
