# Supplementary material for: Design and deployment of the STEEER-AF trial to evaluate and improve guideline adherence: a cluster-randomized trial by the European Society of Cardiology and European Heart Rhythm Association
Source: Europace. 2024 Jun 28;26(7):euae178. doi: 10.1093/europace/euae178 (PMC11289729; doi:10.1093/europace/euae178)
Supplement: euae178_Supplementary_Data [file euae178_supplementary_data.pdf]

Design and deployment of the STEEER-AF trial to evaluate and improve guideline adherence: A cluster-randomised trial by the European Society of Cardiology and European Heart Rhythm Association

SUPPLEMENTAL FILES

ONLINE TABLE S1: THE STEEER-AF TEAM 2

ONLINE TABLE S2: ETHICAL APPROVALS 6

ONLINE TABLE S3: STEEER-AF OUTCOMES 7

ONLINE FIGURE S1: STEEER-AF INTERVENTION 9

ONLINE FIGURE S2: STROKE PREVENTION ALGORITHM – PART 1 10

ONLINE FIGURE S3: STROKE PREVENTION ALGORITHM – PART 2 14

ONLINE FIGURE S4: RHYTHM CONTROL ALGORITHM 18

ONLINE FIGURE S5: KEY MESSAGES OF THE STEEER-AF PROGRAMME 23

ONLINE TABLE S1: THE STEEER-AF TEAM

**Trial Steering Committee**	
Chief Investigators	Dipak Kotecha (University of Birmingham, UK); Isabelle Van Gelder (University Medical Centre Groningen, Netherlands)
National Coordinators	France: Serge Boveda (Clinique Pasteur Toulouse); Germany: Philipp Sommer (Ruhr-Universität Bochum); Italy: Giuseppe Boriani (University of Modena and Reggio Emilia); Poland: Maciej Sterliński (Institute of Cardiology Warsaw); Spain: Lluis Mont; Eduard Guasch (University of Barcelona); United Kingdom: Kim Rajappan (Oxford University Hospitals NHS Trust)
**Coordinating members**	
Methodology and statistics	Chris Gale (University of Leeds, UK); Samir Mehta (Birmingham Clinical Trials Unit, UK); Yongzhong Sun (Birmingham Clinical Trials Unit, UK)
Deployment support	Karina Bunting (University of Birmingham, UK); Colinda Van Deutekom (University Medical Centre Groningen, Netherlands)
Trial coordination	Sandrine Anglars; Celine Arsac; Isabel Bardinet; Clara Berlé; Caroline Bennett; Gabrielle Bonneville; Laura Courleux; Aoife Delmas; Quentin Escartin; Audrey Esperrou Surrel; Adham Gharieb; Christine Gouillard; Anas Islah; Line Joubert; Nathalie Lezer; Vanessa Meyen; Liudmyla Protsiuk; Jean-François Riffaud; François Serrano; Emilie Soriano; Valentina Tursini (European Heart House, France).
**Oversight Committees**	
Strategic Oversight Committee	Chair: Hein Heidbuchel (EHRA).<br>Members: Stephan Achenbach (ESC); Wolfram Döhner (ESC Council on Stroke); Bernard Iung (ESC EORP); Paulus Kirchhof (ESC Education); Susanna Price (ESC Education); Helmut Pürerfellner (EHRA).<br>Past members: Barbara Casadei (ESC); John Camm (EHRA); Christophe Leclercq (EHRA); Bogdan Popescu (ESC EORP).
Data Monitoring Committee	Chair: Alexander Lyon (Imperial College London).<br>Members: Winston Banya – Independent Statistician (Royal Brompton Hospital, London); Robert Hatala (National Cardiovascular Institute NUSCH, Slovak Republic); Pekka Raatikainen (Tampere University Hospital, Finland).
**Content advisors**	
Stroke prevention & rhythm control	Tom De Potter (OLV Hospital Aalst, Belgium); Tatjana Potpara (Clinical Centre of Serbia, Serbia); Thompson G Robinson (University of Leicester, UK).
Patient & public involvement	Mary Stanbury (West Midlands, UK); Truddie Lobban MBE (Arrythmia Alliance, UK).
**National Trainers**	
France	Pierre Baudinaud (European Hospital Georges Pompidou); Pascal Defaye (University Hospital Grenoble Alpes); Eloi Marijon (European Georges Pompidou Hospital).
Germany	Simon Kochhaeuser (Marienhospital); Ursula Rauch Charité (Universitätsmedizin Berlin); Moritz Sinner (LMU University Hospital, LMU Munich).
Italy	Igor Diemberger (University of Bologna); Marco Proietti (University of Milan); Vincenzo Russo (University of Campania "Luigi Vanvitelli" - Monaldi Hospital).

Poland	Pawel Balsam (Warsaw Medical University); Piotr Buchta (Medical University of Silesia); Stanislaw Tubek (Wroclaw Medical University).
Spain	Eusebio García-Izquierdo (Puerta de Hierro University Hospital); Jose Guerra (Hospital de la Santa Creu i Sant Pau, IR SANT PAU, CIBERCV, Universitat Autònoma de Barcelona); Ivo Roca Luque (Cardiovascular Institute. Hospital Clinic, University of Barcelona).
United Kingdom	Mark Davies (Oxford University Hospitals NHS Foundation Trust; Milton Keynes University Hospital NHS Foundation Trust); Afzal Sohaib (St Bartholomew's Hospital); Dewi Thomas (Swansea Bay University Health Board).
**Principal Investigators**	
France	Marine Arnaud; Frédéric Anselme; Rim El Bouazzoui; Andrea Cianci; Christian De Chillou; Maxime De Guillebon; William Escande; Estelle Gandjbakhch; Fabien Garnier; Rodrigue Garcia; Charles Guenancia; Karim Hasni; Nicolas Lellouche; Baptiste Maille; Jean-Philippe Maury; Olivier Piot; Dominique Pavin; Frédéric Sacher; Jérôme Taieb.
Germany	Andreas Bollmann; Martin Borlich; David Duncker; Hans Holger Ebert; Andreas Metzner; Peter Nordbeck; Stefan Spitzer; Dong-In Shin; Roland Tilz; Stephan Willems.
Italy	Matteo Bertini; Giuseppe Boriani; Stefano Fumagalli; Federico Guerra; Jacopo F Imberti; Andrea Mazza; Margherita Padeletti; Antonio Rapacciuolo; Cinzia Valzania.
Poland	Jaroslaw Blicharz; Tomasz Czerski; Robert Gajda; Wiktor Gminski; Adam Gorlo; Monika Lica-Gorzynska; Wojciech Kucejko; Marcin Kostkiewicz; Piotr Paczek; Andrzej Skrzynski; Grzegorz Sobieszek; Hanna Wilk-Manowiec.
Spain	Juan Acosta Martinez; Ignasi Anguera; Joaquin Osca Asensi; Felipe Bisbal; Pilar Cabanas; Naiara Calvo; Juan Fernandez Armenta Pastor; Teresa Lozano; Jose L. Merino; Nuria Rivas-Gandara; Emilce Trucco.
United Kingdom	Mattew Bates; Richard Ang; Richard Bond; Arif Bhuiyan; Chris Hayes; Manish Kalla; Malcolm Finlay; Matthew Lovell; Shawn Morais; Fu Siong Ng; Michala Pedersen; Dan Raine; Bhavesh Sachdev; Norman Qureshi.
**Other supporting staff**	
	Yann Allali (France); Asgher Champsi (United Kingdom); Thomas Deneke (Germany); Kaitlyn Greeley (France); Benoît Guy-Moyat (France); Mikael Laredo (France); Alastair Mobley (United Kingdom); Maximina Ventura (United Kingdom); Andrea Venturelli (Italy).
**Investigators**	
France	Yann Allali; Samuel Ardois; Hugues Bader; Ronan Bakdi; Jérémie Barraud; Valentin Barre1; Fabienne Bellarbre; Samia Benchekroun; Lucas Berthelemy; Sabine Berthier; Nicolas Bidegain; Cédric Bierme1; Caroline Birgy; Laurine Blasi; Jérôme Bouet; Mathilde Bougault; Benjamin Bouyer; Bérengère Cador-Rousseau; Théo Caillol; Marion Charton; Corentin Chaumont; Charlotte Cohen; Marine Dauvergne; Guillaume De Ciancio; Cindy Delangle; Emilie Doche; Mathieu Echivard; Paul-Emile Favre; David Fouassier; Iustina Gaman; Benoît Guy-Moyat; Néfissa Hammache; Chiara Hernandez; Tatiana Hertzog; Sophie Ismael; Ilies Jaballah; Amélie Jamet; Christian Kassasseya; Matthias Lamy; Thierry Laperche; Mikael Laredo; Gabriel Laurent; Paul Le Dantec; Isabelle Lecardonnel; Camille Lepart; Antoine Lepillier; Isabelle Magnin-Poull; Jade Makke; Franck Mandel; Jean-Paul Nghiem; Virginia Nguyen; Marjorie Niro; Charles Nogarede; Arnaud Olivier; Sethi Ouandji; Paul Puie; Abdessamie Rahmouni; Paul Rassinoux; Jerome Razanamahery; Jean-François Riviere;

	Thomas Roxburgh; Arnaud Savoure; Marie Saydi; Jerome Schwartz; Elena Seder; Mathieu Seguret; Matthieu Seguret; Jean-Marc Sellal; Marine Taranzano; Emmanuel Tigier; Romain Tixier; Lilith Tovmassian; Julie Vincent; Marie Wilkin; Sarah Zeriouh.
Germany	Yannic Abdollah-Zadeh; Ruken Özge Akbulak-Stegli; Omar Anwar; Kevin Bachmann; Oliver Berger; Stephanie Brandt; Liesa Castro; Kowalewski Christopher; Nikolaos Dagres; Olti Daka; Steffen Dannenberg; Senem Demirbas; Jannis Dickow; Patrick Dilk; Julia Domagalla; Joerg Eiringhaus; Elena Ene; Florian Fahr; Nele Gene; Jan Gesenberg; Susann Groschke; Tobias Gulde; Melanie Gunawardene; Margarete Heinrichs; Henrike Hilmann; Uta Hoffmann; Stephan Hohmann; Lani Huang Phan; Abu Hussein; Klaus Ingerfurth; Mario Jularic; Eva-Maria Jungclaus; Katrin Kartuchin; Sopio Kavtaradze; Alexander Kinnen; Hanna Kiskalt; Katrin Koch; Lara Kolk; Matthias Korn; Nils Kraus; Kolja Lau; Frank Lindemann; Octavian Maniuc; Mateo Marin-Cuartos; Johanna Mueller-Leisse; Julian Müller; Michelle Müller; Jonas Müntze; Sotirios Nedios; Karin Nentwich; Moritz Nies; Christian Nolte; Julius Obergassel; Annegret Otto; Kobra Pedrood; David Petrich; Jan Reiss; Johannes Rotta Detto Loria; Floran Sahiti; Stephanie Sauer; Julianamin Schäffer; Christian Schneider; Fabian Schuster; Timm Seewöster; Anas Shihab; Clara Stegmann; Johannes Stein; Gunter Stenzel; Würger Tilman; Darko Trajanoski; Sarah Walther; Dong Wang; Manuel Wegner; Simon Julius Winkelmann; Ferdinand Witt; Justinas Zygis.
Italy	Vincenza Abbate; Antonella Accietto; Lucio Addeo; Michele Alfieri; Alessio Aloisio; Domenico Angellotti; Lucia Argento; Matteo Armillotta; Gaetano Asile; Elena Baietti; Cristina Balla; Gianmarco Bastianoni; Sara Belleggia; Maria Grazia Bendini; Diem Berger; Niccolò Bettoni; Chiara Birtolo; Alberto Boccadoro; Felice Borrelli; Leonardo Brugiatelli; Federica Capuani; Simona Chiusolo; Elisa Ciani; Giacomo Cinelli; Maria Grazia Cirigliano; Lucia Ciucciatelli; Francesca Coraducci; Francesca Coretti; Ginevra Corti; Luca Di Ienno; Serena Di Maria; Vincenzo Ercolano; Riccardo Franci Montorzi; Alice Frangione; Elisa Fratini; Alessandro Frizza; Carlo Fumagalli; Luigi Gerra; Turrin Giada; Antonio Raffaele Labriola; Michele Malagu; Federica Mannucci; Lina Manzi; Francesca Marchetti; Chiara Mari; Giulio Menichini; Davide Montanari; Giuseppe Nicoletti; Emanuela Orsi; Federico Paolini; Silvia Pecchioni; Giulia Perfetti; Valerio Pergola; Elena Perini; Irene Piccinini; Laura Pinton; Giancarlo Ralli; Ilaria Righelli; Irene Ruotolo; Adelina Selimi; Fiorenzo Simonetti; Giulia Stronati; Nicole Suma; Gaetano Todde; Paolo Tofoni; Lorenzo Torselletti; Virginia Tortu; Yari Valeri; Andrea Venturelli; Nicola Verde; Simona Virciglio; Francesco Vitali; Marco Vitolo.
Poland	Karolina Adamczyk; Wiesław Banaś; Marcin Barzak; Piotr Barzak; Krzysztof Bielamowicz; Alicja Borowska; Maciej Cholewinski; Karina Chorazewicz; Robert Ciesielski; Krzysztof Cieślik; Paulina Deleskiewicz; Lukasz Deptula; Agata Donczyk; Natalia Dudzinska; Wojciech Dziadek; Agnieszka Fabisiak; Tomasz Fiderewicz; Agnieszka Fil; Ryszard Gluszczyk; Pawel Harpula; Jewgienija Holovatska; Anna Janiszewska; Bogdan Janus; Michal Jasionka; Maria Jaworska-Drozdowska; Sylwia Jedrej; Katarzyna Jutkiewicz; Jacek Karasinski; Artur Kobus; Daria Koszelnik; Aneta Kowalczyk-Rutkowska; Tomasz Kozioł; Joalnata Krupa; Monika Kujawiak; Paulina Kulczyńska; Barbara Kwiatkowska; Iwona Lagoda; Monika Landzberg; Magdalena Lewandowska; Michal Miszczak; Krystyna Moskal; Małgorzata Niedzielska; Marcin Oglodek; Bartosz Olajossy; Agnieszka Pasiowiec; Artur Rok; Sylwia Sawicka; Grzegorz Skonieczny; Marta Sondej; Dorota Stańczuk; Mikolaj Strakowski; Jacek Szalewski; Rafal Szewczuk; Piotr Szubielski; Anna Szwiec; Natalia Wasilewska; Paulina Wierzbinska-Kornek; Rafal Witkowski; Emilia Zablocka; Alicja Zajac Lesniak; Kamil Zenderowski.
Spain	Laia Alcober; Laura Álvarez Roy; Eduardo Arana Rueda; Antonio Aranda Dios; Julia Aranyo; Emilio Arbas Redondo; Santiago Aviles; Hebert David Ayala More;

	Josefa Azpiroz Franch; Anna Bardalet; Victor Bazán; Eva Benito; Clara Bergua Martínez; Domingo Bleda Fernandez; Daniel Bosch Portell; Ramón Brugada; Ascension Cabrera Santos; Ana María Campos Pareja; Dolores Cañadas Pruaño; Fermín Cancio González; Berenice Caneiro-Queija; Javier Cantalapiedra Romero; Sergio Castrejon Castrejon; María Cespón-Fernández; Alfredo Chauca; David Chivite; Edurad Claver; Rafael José Cobas-Paz; Juan Caro Codon; Javier Conejos; Raquel Cruz; Cristina De Prado La Cueva; Maria Del Mar Ras; Cristina Domenech; Eva Domingo Baldrich; Diana Domingo Valero; Pablo Domínguez Erquicia; Luis Manuel Domínguez-Rodríguez; Carlos Escobar Cervantes; Begoña Espinosa; Valentina Faga; Magda Muelas Fernandez; Marta Florensa; Antia Fraga; Manuel Frutos; Manuel Frutos López; Tamara García López; Lorena García Riesco; Carlos González Guerrero; Antonio Grande Trillo; Leonardo Guido; Lorena Herrador Galindo; Angel Iniesta Manjavacas; Carlos Labata; María Lasala Alastuey; Ana Ledo-Piñeiro; Markus Linhart; Andrea Lizancos-Castro; Pascual Llongueras Espi; Adriana Lloret; Judith Llussa; Carlos Lopez Menchero; Carlos Rubén López Perales; Pablo Jordan Marchite; Juan Gabriel Martínez; Marcel Martínez Cossiani; Ricardo Martínez Picazo; Julia Martínez Solé; Daniel Bartolomé Mateos; Paloma Menéndez Polo; Jordi Merce; Asier Molinero; Isabel Muñoz Pousa; Javier Navarrete; Vanesa Noriega-Caro; Inmaculada Noval Morillas; Aleix Olivella; Víctor Ortiz Martínez; José Antonio Parada-Barcia; Julia Pascual; Julia Pedraza; Víctor Pérez Roselló; Mar Piedecausa; Ana Maria Ponce Lopez; Marta Pons; Isabel Dolores Poveda Pinedo; Majda Radaidan Hazzaoui; Juan Ramon Rey; Diego Rangel Sousa; Sergio Raposeiras-Roubín; Pau Rello Sabaté; Julian Rodriguez Garcia; Jose Angel Rodriguez Mariscal; Jesús Rodríguez Silva; Elvira Ruiz Blasco; Monica Ruiz Pombo; Diego Mialdea Salmerón; Andrea Salvador Catalán; Juan Sánchez Brotons; Susana Sanchez Fernández; Jose Carlos Sánchez Martínez; Jesús Sánchez Pardo; Monica Sanchez Picchio; Alba Santos Ortega; Jordi Serra; Silvia Serrano; Alicia Serrano Romero; Manjot Singh; Toni Soriano; Jose Antonio Sorolla Romero; Simón Tapia Mahan; Daniel Tebar Marquez; Blanca Torres; Filipa Valente; Beatriz Valero; Nuria Vallejo; María Vidal; Roger Villuendas; Perla Yupanqui Sandoval.
United Kingdom	Zeina Abu Orabi; Christine Acourt; Emmanuel Ako; Thanusha Ananthakumar; Catherine Atkin; Sveeta Badiani; Charles Badu-Boateng; Hester Baverstock; Christos Bourantas; Claire Brookes; Eden Cabucos; Mike Chapman; Jibi Cheriyan; Chern Hsiang Choy; Rebecca Coleman; Louise Collier; Michael Crisp; Hannah Cunningham; Kristi Davies; Rodney De Palma; Nicola De Savary; Flavio Gil Lopes De Sousa; Ameer Al-Bassam Dhanuka Perera; Rui Da Silva Dias; Nneka Dike; Ayse Djahit; Subhabrata Dutta; Smita Dutta-Roy; Ayman Emam; Mihaela Ene; Sabeena Eydatoula; Charlie Gardiner; Oghenekome Gbinigie; Manju George; Christopher Goode; Caroline Gourlay; Jennifer Holland; Sebastian Horne; Sooraya Housee; Sarah Hughes; Morna Johnston; Nick Jones; Mark Jordan; Raffi Kaprielian; Jan Keenan; Sadia Khan; Sarah Kingham; Hayley Kingwell; Jamie Kitt; Rafail Kotronias; Siddarth Kumar; Nicola Bowers Lamprini Kirkineska; Meher Lehri; Cheryl Livingtone; Angelica Lotito; Tin Lwin; Chenhan Sam Ma; Sarah Mcgrath; Helen Mclennan; Anthony Mechery; Ben Mitchell; Vicky Moore; Angela Murphy; Duduzile Musa; Szymon Musiol; Kiruba Nagaratnam; Helen Nolte; Teresa O'nwere-Tan; Amanda Pugsley; Tony Rice; Saad Saeed; Aureo Sanz-Cepero; Rhona Schwartz; Nihit Shah; M. Shanmugawaiha; Shaima Shareef; Abhishek Shetye; Cas Shotter-Weetman; Simar Pal Singh; Pavidra Sivanandarajah; Claire Smith; Neil Stewart; Sheena Sukumaran; Ashley Theakston; Nicol Vaidya; Haseeb Valli; Shanti Velmurugan; Victor Voon; Clair Wyatt

EORP = EURObservational Research Programme; EHRA = European Heart Rhythm Association; ESC = European Society of Cardiology.

ONLINE TABLE S2: ETHICAL APPROVALS

Country	Institution	Approval number
France	Comité de Protection des Personnes<br>Est-II<br>Siège : CHRU – Hôpital Saint Jacques	Réf SIRIPH : 20.09.30.40105
Germany	Hamburg Medical Chamber	2021-200011-BO-bet
	Universität Zu Lübeck	20-403
	Universität Leipzig	274/21-1k
	Medizinische Hochschule Hannover	Nr.9774_BO_K_2021
	Schleswig-Holstein Medical Association	EK/AH/EN 052/21 m
	Sächsische Landesärztekammer	EK-BR-59/21-1
Italy	Comitato Etico di Area Vasta Emilia Centro	1285/2020/SPER/AOUMO SIRER ID2030
	Comitato Etico Regionale - CER Umbria	4052/19
	Comitato Etico di Area Vasta Emilia Centro della Regione Emilia-Romagna (CE-AVEC) Ethics	448/2021/Sper/AOUFe
	Comitato Etico Regionale delle Marche	n. cerm 2021 393
	Comitato Etico Universita Federico II	186/21
Poland	Heart Rhythm Disorders Clinic, National Institute of Cardiology	NA
Spain	el Comité de Ética de Investigación con Medicamentos del Hospital Universitario Vall d'Hebron	PR(AG)613/2020
	el Comité de ética de Investigación con medicamentos y comision dCEIM GIRONA	PR(AG)613/2020
United Kingdom	NHS Health Research Authority	21/PR/0040

ONLINE TABLE S3: STEEER-AF OUTCOMES

**Co-primary outcomes**	
Adherence to class I and III ESC guidelines for stroke prevention	Proportion of patients whose therapy adheres fully with these recommendations, including the appropriate prescription of oral anticoagulation in patients with an elevated stroke risk score, appropriate dosing of anticoagulants, avoiding inappropriate prescription of antiplatelet agents and/or oral anticoagulants in patients at low stroke risk, limiting concomitant use of anticoagulant and antiplatelet therapy, and secondary stroke prevention strategies.
Adherence to class I and III ESC guidelines for rhythm control therapy	Proportion of patients whose therapy adheres fully with these recommendations, including symptom-directed approaches, acute cardioversion of AF and associated stroke prevention strategies, antiarrhythmic drug prescription, catheter ablation of AF, AF surgery, and rhythm control in specific patient populations.
**Secondary outcomes**	
Proportion of relevant guidelines adhered to for stroke prevention	Class I and III ESC guidelines adhered to out of all eligible recommendations for that patient.
Proportion of relevant guidelines adhered to for rhythm control	Class I and III ESC guidelines adhered to out of all eligible recommendations for that patient.
Proportion of patients treated with oral anticoagulants including both class I and II guideline indications	As per 2016 and 2020 ESC Guidelines on AF: Women with CHA_2DS_2-VASc score 2 or above; men with CHA_2DS_2-VASc score 1 or above.
Proportion of patients treated with oral anticoagulants according to class I guideline indications	As per 2016 and 2020 ESC Guidelines on AF: Women with CHA_2DS_2-VASc score 3 or above; men with CHA_2DS_2-VASc score 2 or above.
Integrated AF management approach (education, lifestyle support, self-management, shared decision making, support tools, adherence and multidisciplinary management)	Score out of eight for each patient consisting of the following consensus-defined markers of good integrated care (patient and investigator reported):<br>(1) Structured patient education provided on stroke prevention and oral anticoagulant therapy; (2) Advice and education on lifestyle and risk factors management given to patient; (3) Support provided to patients to make lifestyle changes; (4) Encouragement and empowerment of the patient for self-management; (5) Shared decision making approach used; (6) Use of technology tools, checklists or clinical decision support tools; (7) Monitoring of adherence to therapy and effectiveness; (8) Engagement of a multidisciplinary team in AF management.

Patient-reported quality of life	Patient reported using the EQ-5D-5L questionnaire; mean index value and visual analogue scale.
**Process outcomes (intervention arm only)**	
Improvement in knowledge and guideline-adherent practice by healthcare professionals using the educational intervention	(1) Multiple choice questions following the learning objectives of each educational module included in the online intervention; (2) Time spent on the online platform for each learner; (3) Percentage of required reading links clicked by each learner; (4) In-person interaction of the learner with the National Trainer; (5) Commitment to change plan to assess local change in clinical practice.
**Remote follow-up**	
Collation of information on major clinical events that have occurred for each patient since their baseline visit.	Primary remote outcome: Composite of all-cause mortality, non-fatal stroke, transient ischaemic attack, pulmonary embolus, systemic embolic event, acute coronary syndrome, myocardial infarction, hospitalisation for heart failure, and/or major and clinically-relevant non-major bleeding (defined as requiring a hospital admission).<br>Secondary remote outcomes: Individual components of the primary outcome; hospital admissions (number and duration).

The CHA_2DS_2-VASc clinical risk score for stroke and thromboembolism in atrial fibrillation gives 2 points for age ≥75 years and prior stroke, transient ischaemic attack or systematic embolus, and 1 point for chronic heart failure, hypertension, diabetes mellitus, vascular disease, age ≥65 years or female gender.

AF = atrial fibrillation; ESC = European Society of Cardiology.

ONLINE FIGURE S1: STEEER-AF INTERVENTION

Summary of the educational intervention provided to investigators in STEEER-AF centres randomised to the intervention group.

MCQ = multiple choice questions; STEEER-AF = Stroke prevention and rhythm control Treatment: Evaluation of an Educational programme of the European society of cardiology in a cluster-Randomised trial in patients with Atrial Fibrillation; Wk = week of intervention.

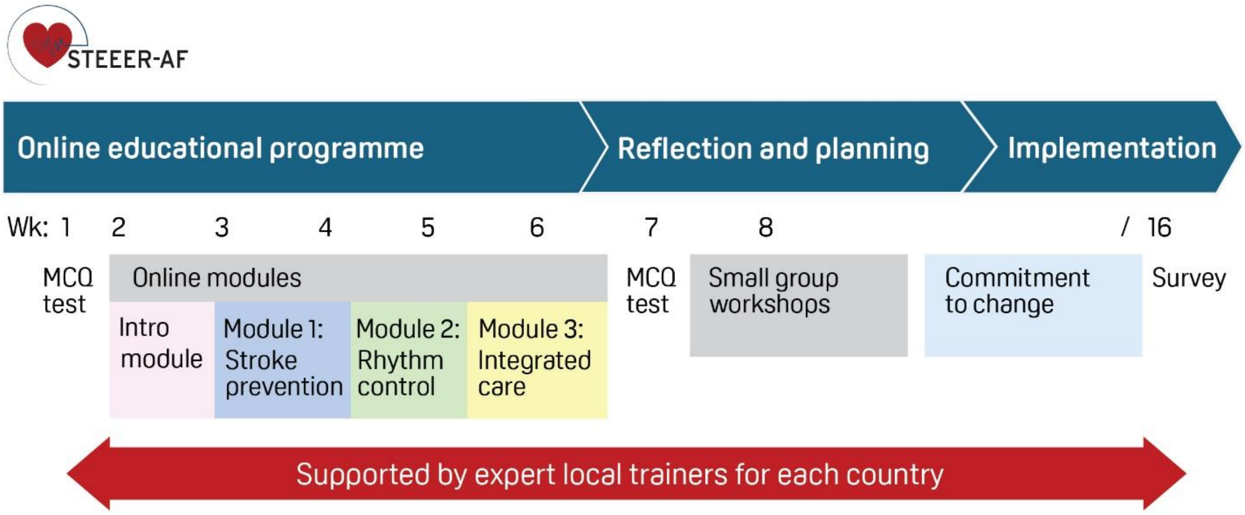

ONLINE FIGURE S2: STROKE PREVENTION ALGORITHM – PART 1

Algorithm to determine patient-level adherence to class I and III European Society of Cardiology guideline recommendations for stroke prevention in women with a CHA_2DS_2-VASc score <3 and men <2. The CHA_2DS_2-VASc clinical risk score for stroke and thromboembolism in atrial fibrillation gives 2 points for age ≥75 years and prior stroke, transient ischaemic attack or systematic embolus, and 1 point for chronic heart failure, hypertension, diabetes mellitus, vascular disease, age ≥65 years or female gender.

AF = atrial fibrillation; APTT = activated partial thromboplastin clotting time; DOAC = direct oral anticoagulant; eGFR = estimated glomerular filtration rate; INR = international normalised ratio for prothrombin time; N = no; PCI = percutaneous coronary intervention; TIA = transient ischaemic attack; TTR = time in therapeutic range; VKA = vitamin K antagonist oral anticoagulant; Y = yes.

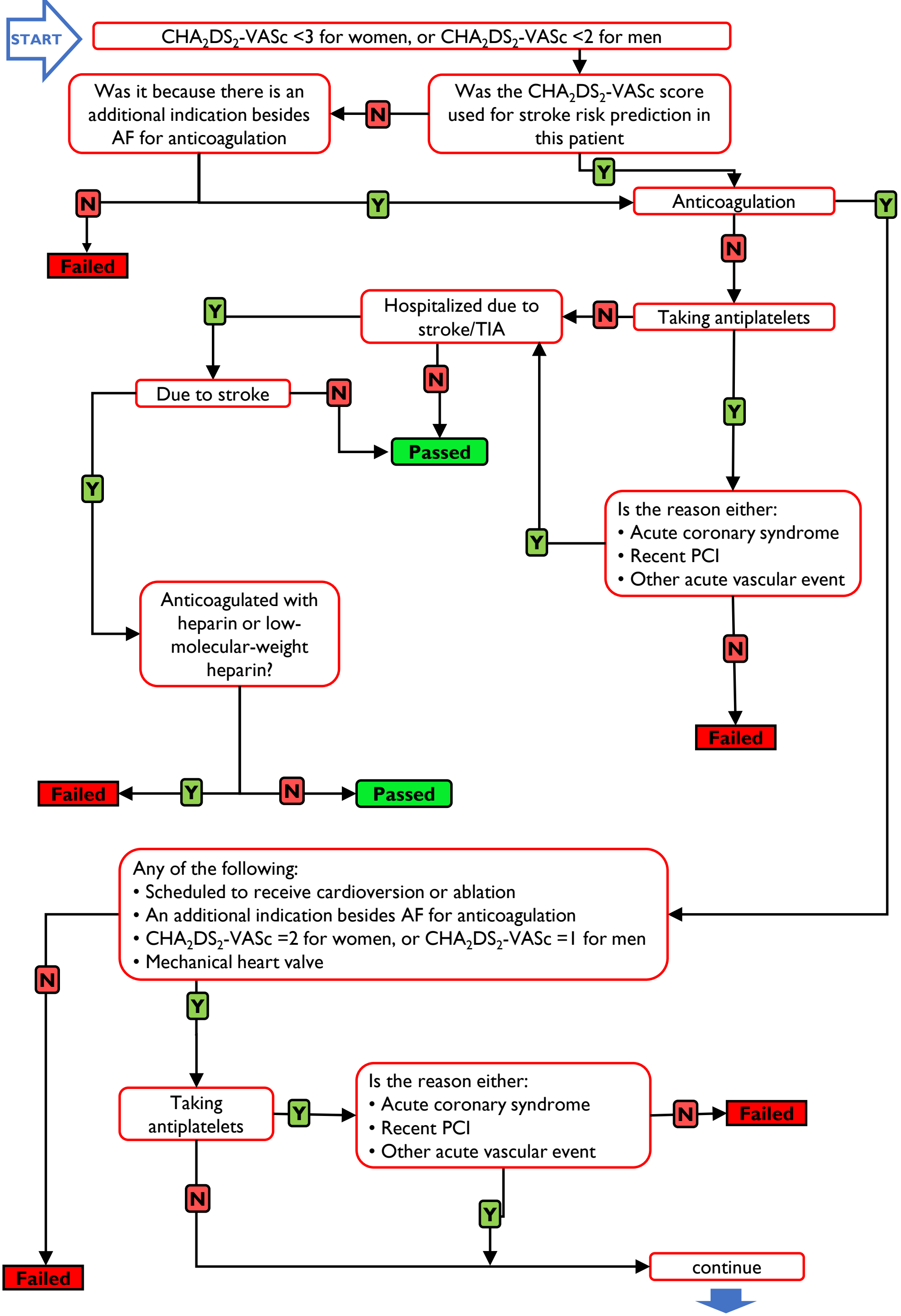
START
CHA2DS2-VASc <3 for women, or CHA2DS2-VASc <2 for men
Was the CHA2DS2-VASc score used for stroke risk prediction in this patient
N
Was it because there is an additional indication besides AF for anticoagulation
Y
N
Failed
Y
Anticoagulation
Y
N
Taking antiplatelets
N
Hospitalized due to stroke/TIA
Y
Due to stroke
N
N
Passed
Y
Y
Is the reason either:
• Acute coronary syndrome
• Recent PCI
• Other acute vascular event
Anticoagulated with heparin or low-molecular-weight heparin?
N
Failed
Failed
Y
N
Passed
Any of the following:
• Scheduled to receive cardioversion or ablation
• An additional indication besides AF for anticoagulation
• CHA2DS2-VASc =2 for women, or CHA2DS2-VASc =1 for men
• Mechanical heart valve
N
Y
Taking antiplatelets
Y
Is the reason either:
• Acute coronary syndrome
• Recent PCI
• Other acute vascular event
N
Failed
N
Y
Failed
continue

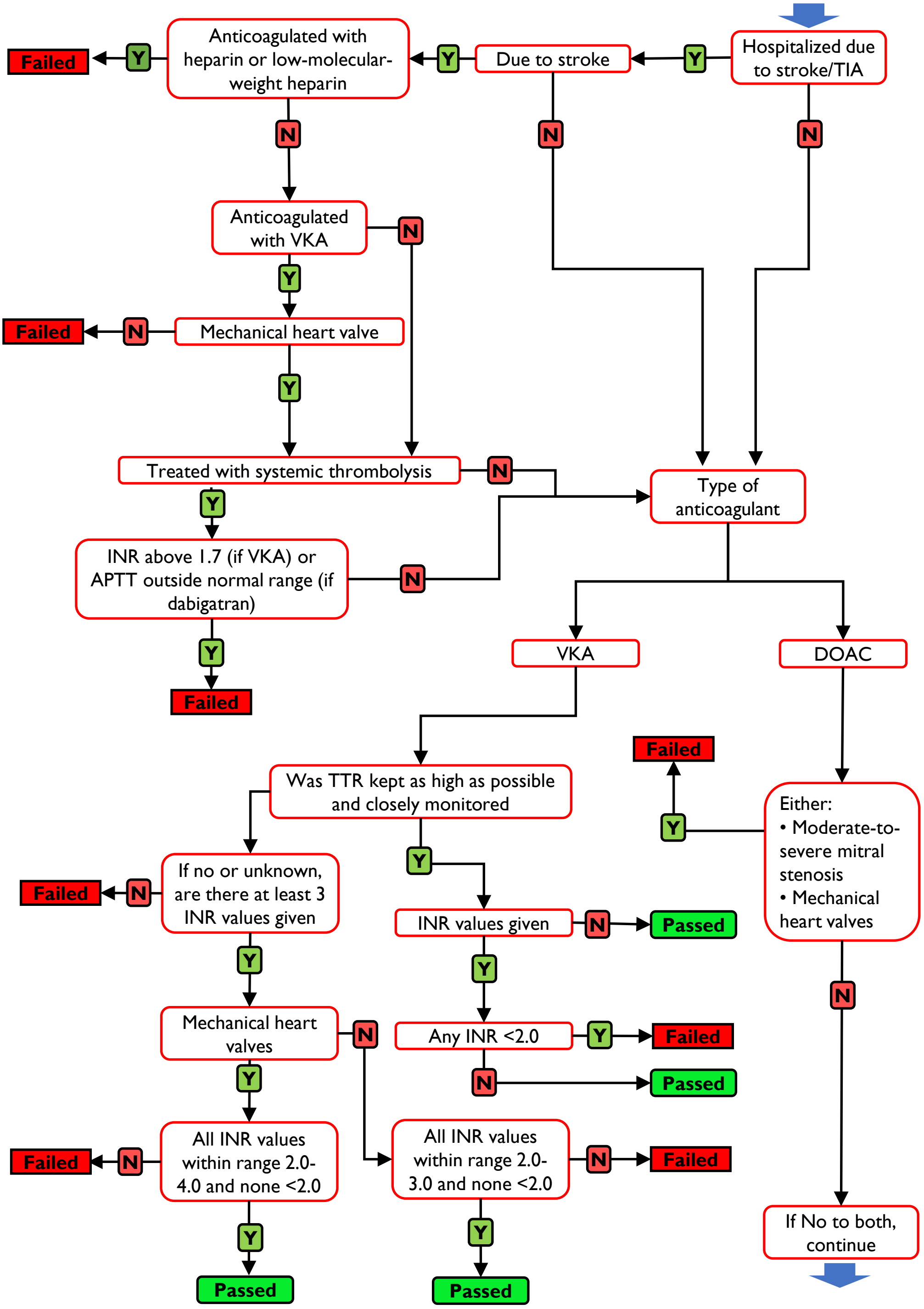

Hospitalized due to stroke/TIA
Y
N
Due to stroke
Y
N
Anticoagulated with heparin or low-molecular-weight heparin
Y
Failed
N
Anticoagulated with VKA
Y
N
Mechanical heart valve
N
Failed
Y
Treated with systemic thrombolysis
N
Y
INR above 1.7 (if VKA) or APTT outside normal range (if dabigatran)
N
Y
Failed
Type of anticoagulant
VKA
DOAC
Was TTR kept as high as possible and closely monitored
If no or unknown, are there at least 3 INR values given
N
Failed
Y
Mechanical heart valves
Y
N
All INR values within range 2.0-4.0 and none <2.0
N
Failed
Y
Passed
Y
INR values given
N
Passed
Y
Any INR <2.0
Y
Failed
N
Passed
All INR values within range 2.0-3.0 and none <2.0
N
Failed
Y
Passed
Either:
• Moderate-to-severe mitral stenosis
• Mechanical heart valves
Y
Failed
N
If No to both, continue

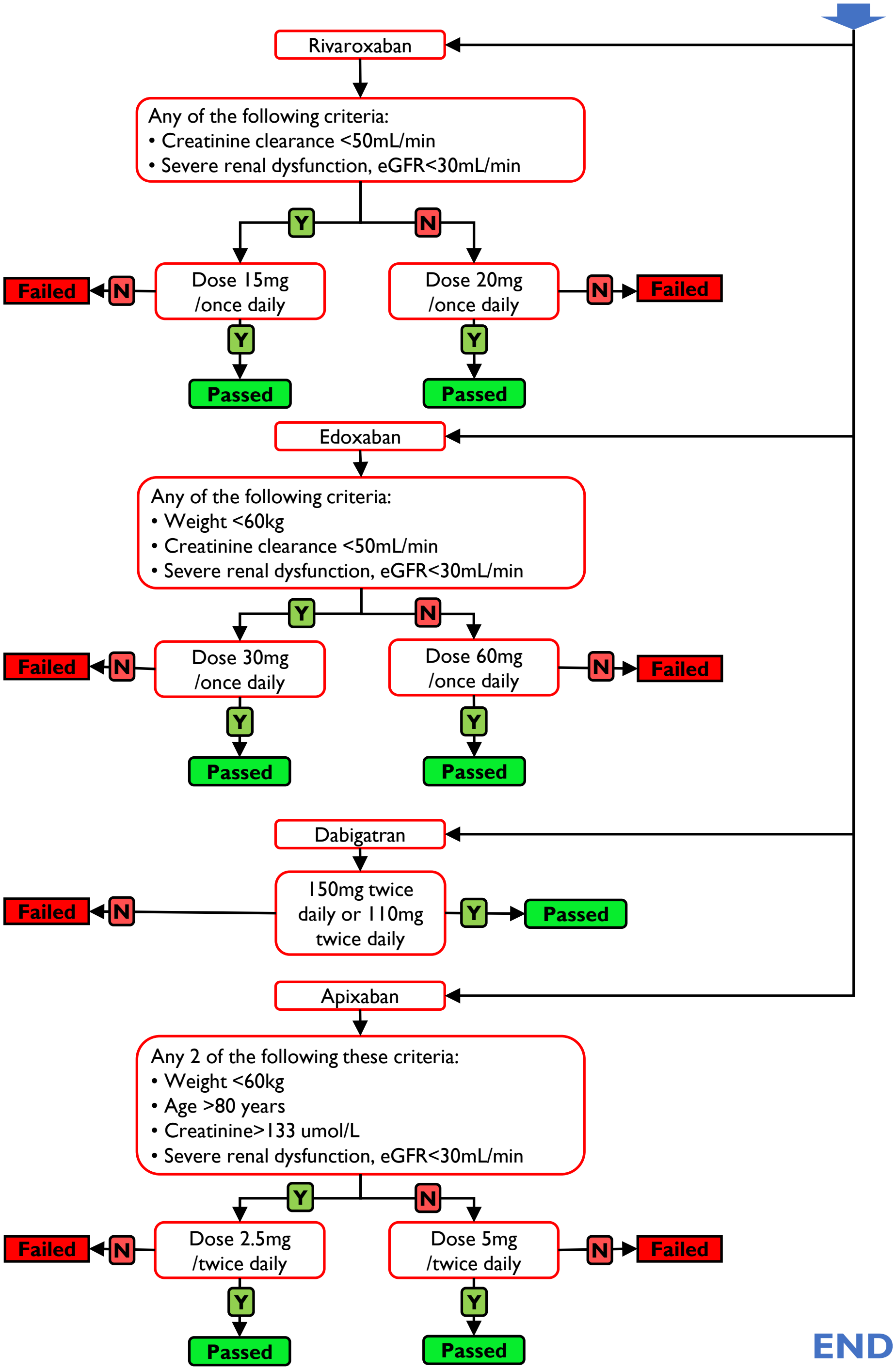
Rivaroxaban
Any of the following criteria:
• Creatinine clearance <50mL/min
• Severe renal dysfunction, eGFR<30mL/min
Y
N
Dose 15mg /once daily
Dose 20mg /once daily
Failed
N
N
Failed
Y
Y
Passed
Passed
Edoxaban
Any of the following criteria:
• Weight <60kg
• Creatinine clearance <50mL/min
• Severe renal dysfunction, eGFR<30mL/min
Y
N
Dose 30mg /once daily
Dose 60mg /once daily
Failed
N
N
Failed
Y
Y
Passed
Passed
Dabigatran
150mg twice daily or 110mg twice daily
Failed
N
Y
Passed
Apixaban
Any 2 of the following these criteria:
• Weight <60kg
• Age >80 years
• Creatinine>133 umol/L
• Severe renal dysfunction, eGFR<30mL/min
Y
N
Dose 2.5mg /twice daily
Dose 5mg /twice daily
Failed
N
N
Failed
Y
Y
Passed
Passed
END

ONLINE FIGURE S3: STROKE PREVENTION ALGORITHM – PART 2

Algorithm to determine patient-level adherence to class I and III European Society of Cardiology guideline recommendations for stroke prevention in women with a CHA_2DS_2-VASc score ≥3 and men ≥2. The CHA_2DS_2-VASc clinical risk score for stroke and thromboembolism in atrial fibrillation gives 2 points for age ≥75 years and prior stroke, transient ischaemic attack or systematic embolus, and 1 point for chronic heart failure, hypertension, diabetes mellitus, vascular disease, age ≥65 years or female gender.

AF = atrial fibrillation; APTT = activated partial thromboplastin clotting time; DOAC = direct oral anticoagulant; eGFR = estimated glomerular filtration rate; INR = international normalised ratio for prothrombin time; LAA = left atrial appendage; N = no; PCI = percutaneous coronary intervention; TIA = transient ischaemic attack; TTR = time in therapeutic range; VKA = vitamin K antagonist oral anticoagulant; Y = yes.

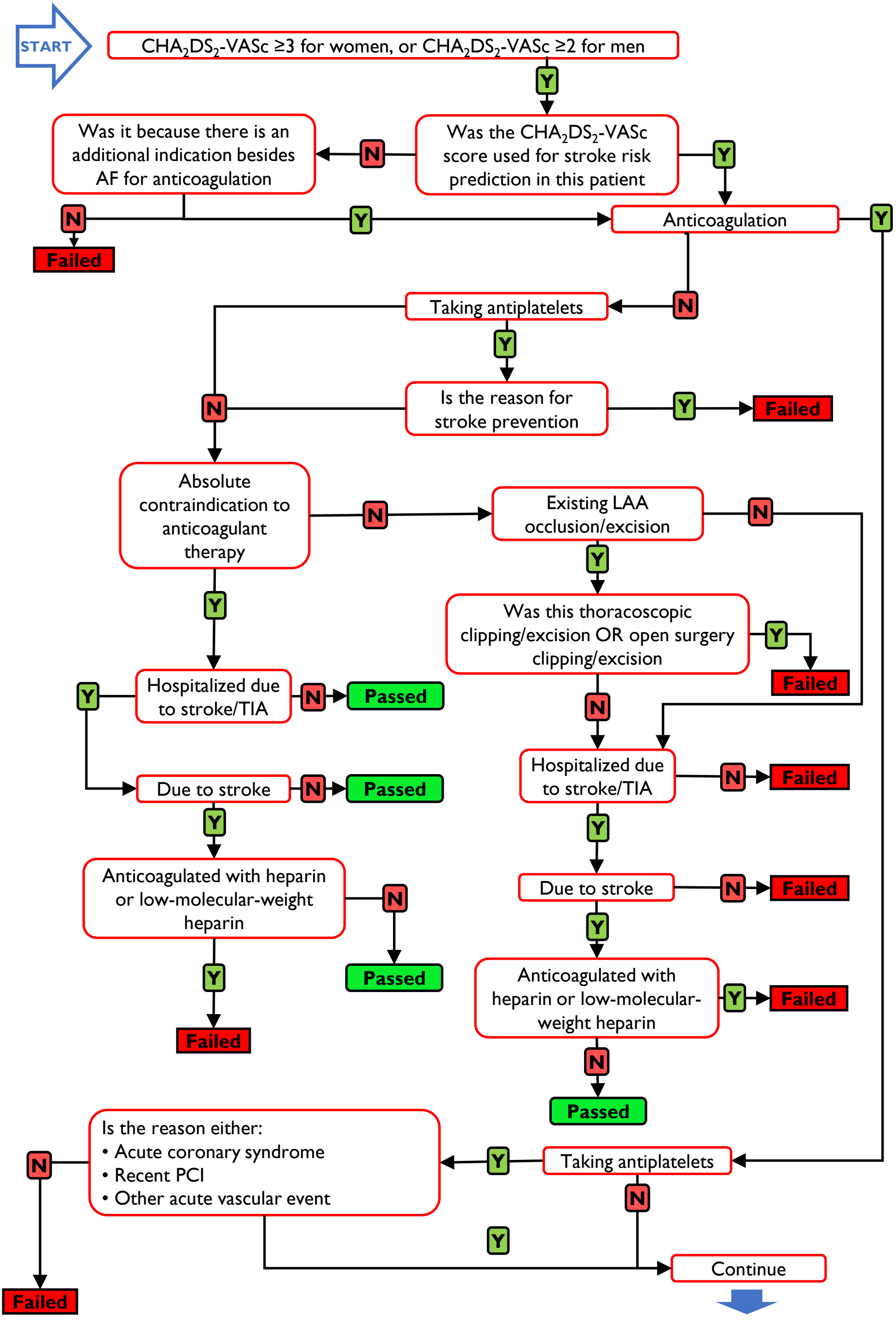
START
CHA2DS2-VASc ≥3 for women, or CHA2DS2-VASc ≥2 for men
Was the CHA2DS2-VASc score used for stroke risk prediction in this patient
Was it because there is an additional indication besides AF for anticoagulation
Failed
Anticoagulation
Taking antiplatelets
Is the reason for stroke prevention
Failed
Absolute contraindication to anticoagulant therapy
Existing LAA occlusion/excision
Was this thoracoscopic clipping/excision OR open surgery clipping/excision
Failed
Hospitalized due to stroke/TIA
Passed
Due to stroke
Passed
Anticoagulated with heparin or low-molecular-weight heparin
Passed
Failed
Hospitalized due to stroke/TIA
Failed
Due to stroke
Failed
Anticoagulated with heparin or low-molecular-weight heparin
Failed
Passed
Is the reason either:
• Acute coronary syndrome
• Recent PCI
• Other acute vascular event
Taking antiplatelets
Failed
Continue
Y
N

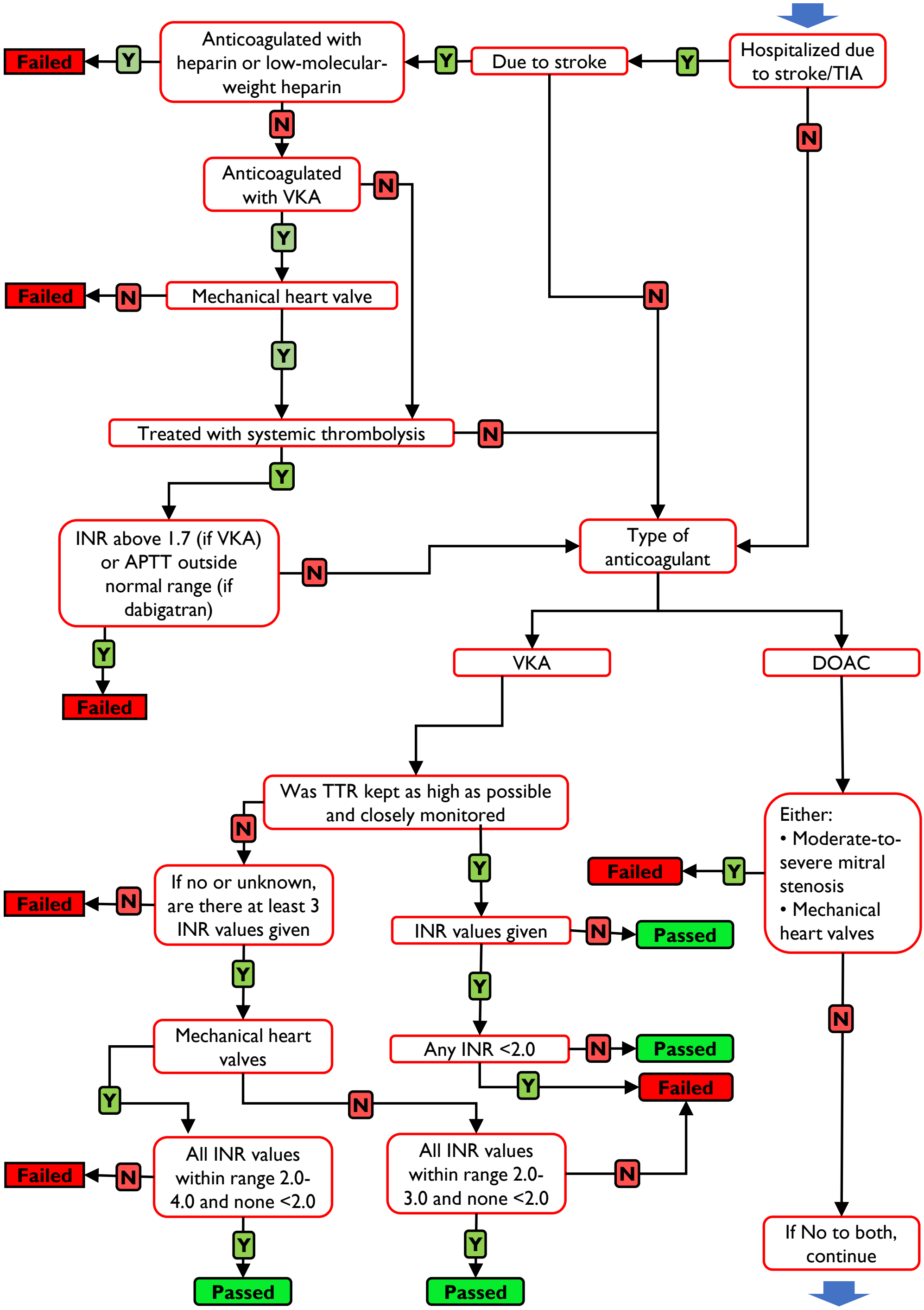

Hospitalized due to stroke/TIA
Due to stroke
Anticoagulated with heparin or low-molecular-weight heparin
Failed
Anticoagulated with VKA
Mechanical heart valve
Failed
Treated with systemic thrombolysis
INR above 1.7 (if VKA) or APTT outside normal range (if dabigatran)
Failed
Type of anticoagulant
VKA
DOAC
Was TTR kept as high as possible and closely monitored
If no or unknown, are there at least 3 INR values given
Failed
INR values given
Passed
Any INR <2.0
Passed
Failed
Mechanical heart valves
All INR values within range 2.0-4.0 and none <2.0
Failed
Passed
All INR values within range 2.0-3.0 and none <2.0
Passed
Either:
• Moderate-to-severe mitral stenosis
• Mechanical heart valves
Failed
If No to both, continue
Y
N

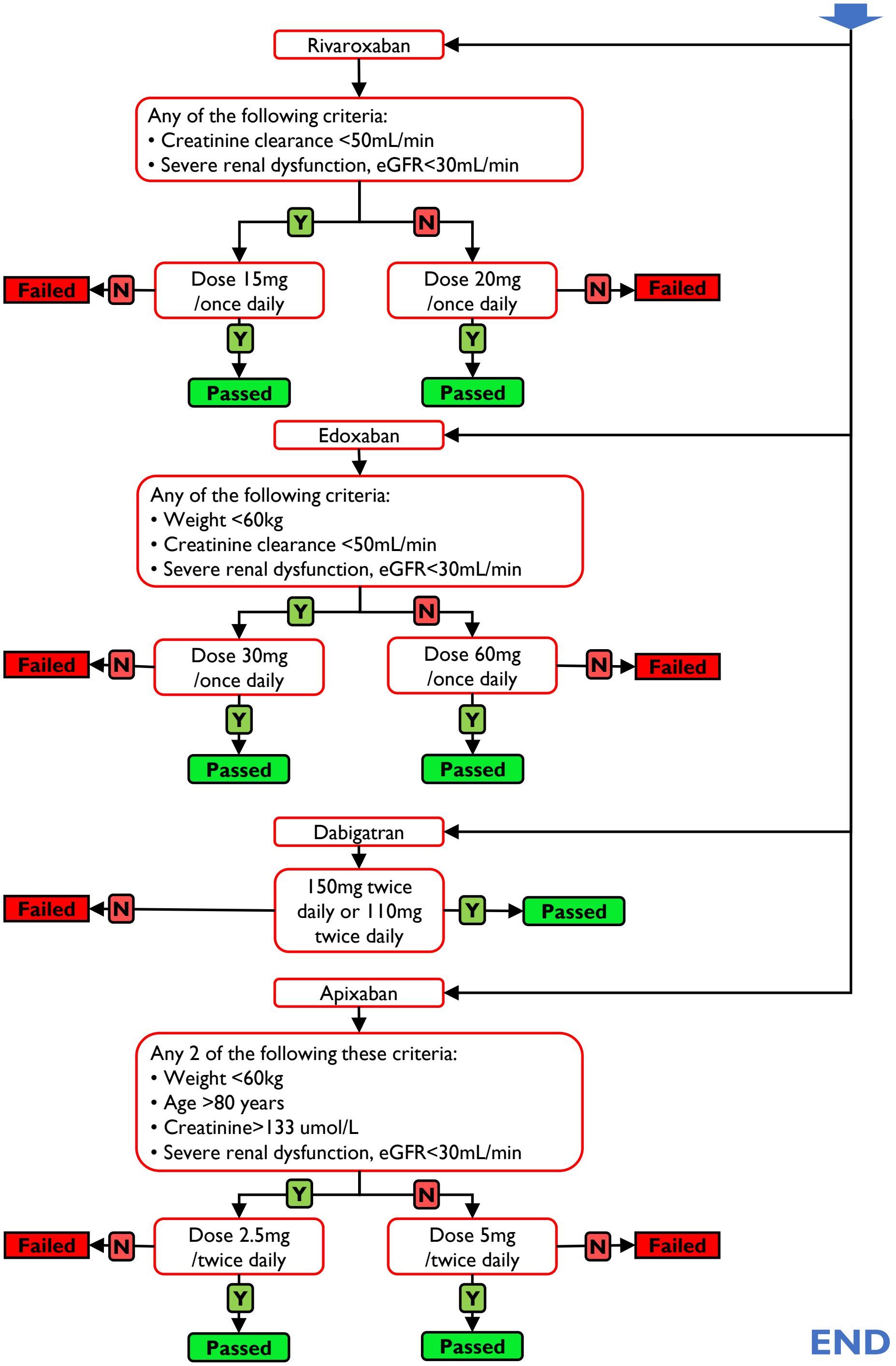
Rivaroxaban
Any of the following criteria:
• Creatinine clearance <50mL/min
• Severe renal dysfunction, eGFR<30mL/min
Y
N
Dose 15mg /once daily
Dose 20mg /once daily
N
Failed
Y
Passed
Edoxaban
Any of the following criteria:
• Weight <60kg
• Creatinine clearance <50mL/min
• Severe renal dysfunction, eGFR<30mL/min
Dose 30mg /once daily
Dose 60mg /once daily
Dabigatran
150mg twice daily or 110mg twice daily
Apixaban
Any 2 of the following these criteria:
• Weight <60kg
• Age >80 years
• Creatinine>133 umol/L
• Severe renal dysfunction, eGFR<30mL/min
Dose 2.5mg /twice daily
Dose 5mg /twice daily
END

ONLINE FIGURE S4: RHYTHM CONTROL ALGORITHM

Algorithm to determine patient-level adherence to class I and III European Society of Cardiology guideline recommendations for rhythm control.

AF = atrial fibrillation; CRT-D = cardiac resynchronisation therapy with defibrillator; CRT-P = cardiac resynchronisation therapy with pacing; EHRA = European Heart Rhythm Association; ICD = implantable cardioverter-defibrillator; LVEF = left ventricular ejection fraction; N = no; OAC = oral anticoagulant therapy; TOE = transoesophageal echocardiogram; Y = yes.

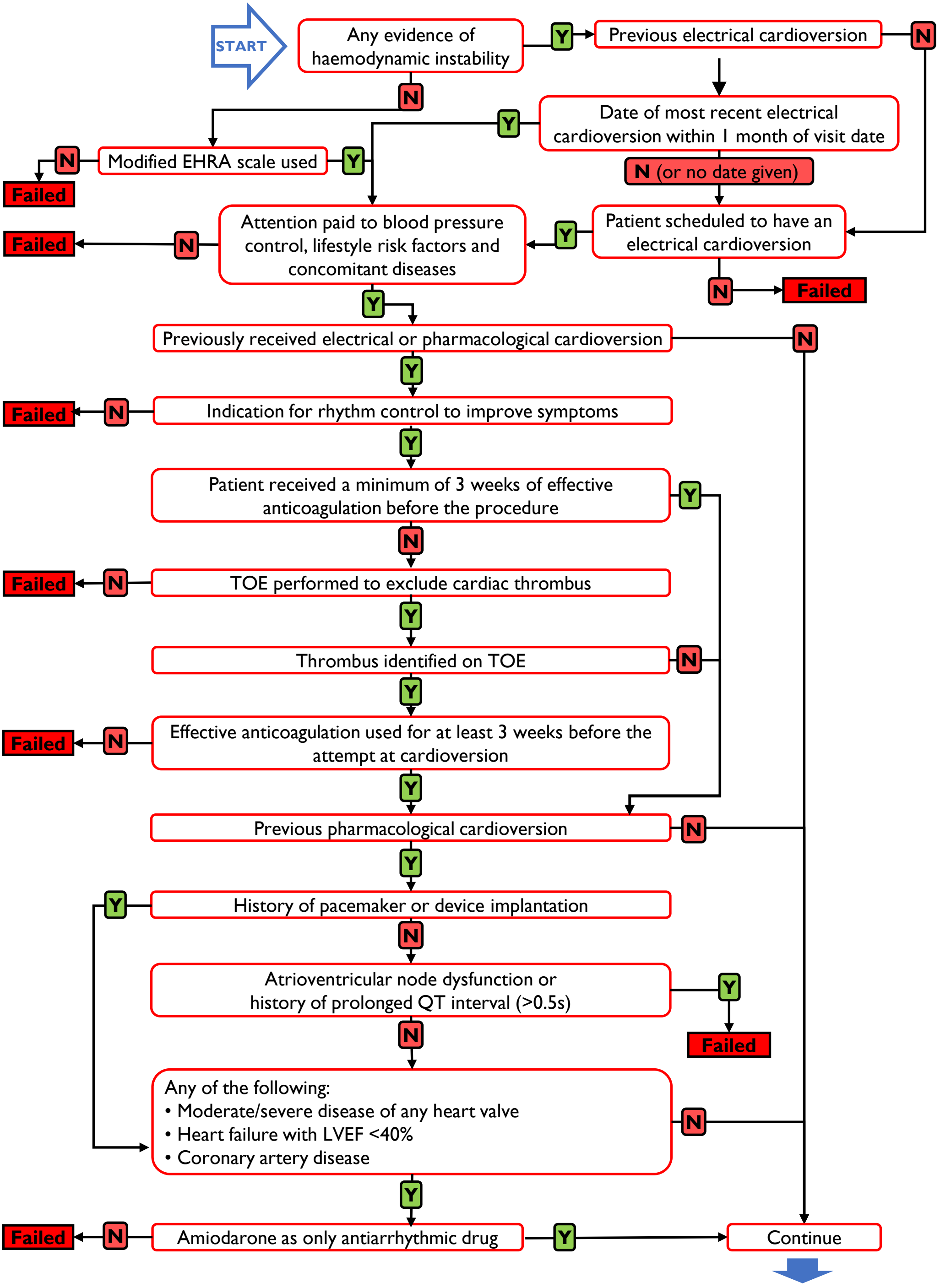
START
Any evidence of haemodynamic instability
Y
N
Previous electrical cardioversion
Date of most recent electrical cardioversion within 1 month of visit date
N (or no date given)
Modified EHRA scale used
Failed
Patient scheduled to have an electrical cardioversion
Attention paid to blood pressure control, lifestyle risk factors and concomitant diseases
Previously received electrical or pharmacological cardioversion
Indication for rhythm control to improve symptoms
Patient received a minimum of 3 weeks of effective anticoagulation before the procedure
TOE performed to exclude cardiac thrombus
Thrombus identified on TOE
Effective anticoagulation used for at least 3 weeks before the attempt at cardioversion
Previous pharmacological cardioversion
History of pacemaker or device implantation
Atrioventricular node dysfunction or history of prolonged QT interval (>0.5s)
Any of the following:
• Moderate/severe disease of any heart valve
• Heart failure with LVEF <40%
• Coronary artery disease
Amiodarone as only antiarrhythmic drug
Continue

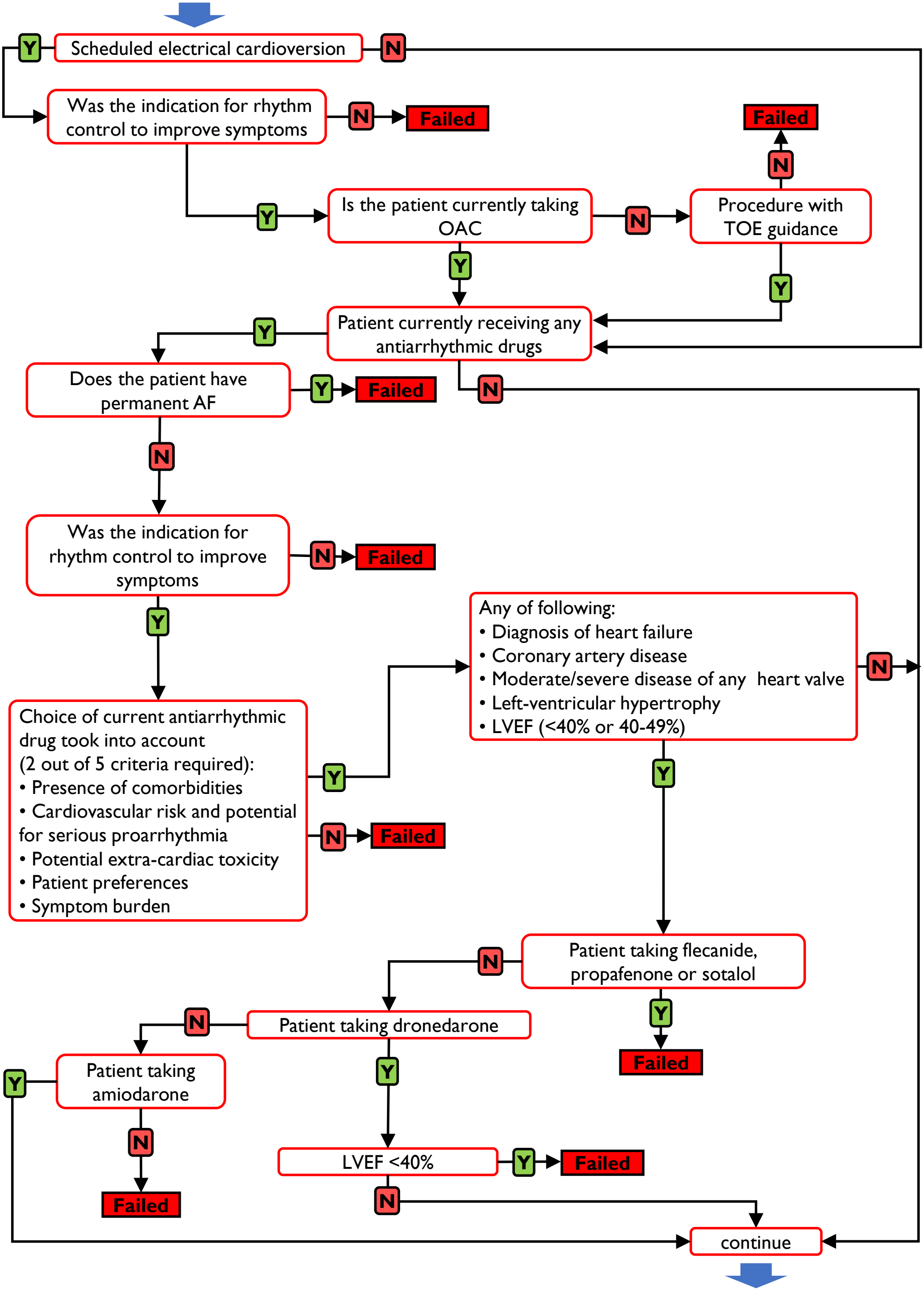
Scheduled electrical cardioversion
Y
N
Was the indication for rhythm control to improve symptoms
N
Failed
Y
Is the patient currently taking OAC
N
Procedure with TOE guidance
N
Failed
Y
Y
Patient currently receiving any antiarrhythmic drugs
Y
N
Does the patient have permanent AF
Y
Failed
N
Was the indication for rhythm control to improve symptoms
N
Failed
Y
Choice of current antiarrhythmic drug took into account (2 out of 5 criteria required):
• Presence of comorbidities
• Cardiovascular risk and potential for serious proarrhythmia
• Potential extra-cardiac toxicity
• Patient preferences
• Symptom burden
Y
N
Failed
Any of following:
• Diagnosis of heart failure
• Coronary artery disease
• Moderate/severe disease of any heart valve
• Left-ventricular hypertrophy
• LVEF (<40% or 40-49%)
N
Y
Patient taking flecanide, propafenone or sotalol
Y
Failed
N
Patient taking dronedarone
Y
LVEF <40%
Y
Failed
N
N
Patient taking amiodarone
Y
N
Failed
continue

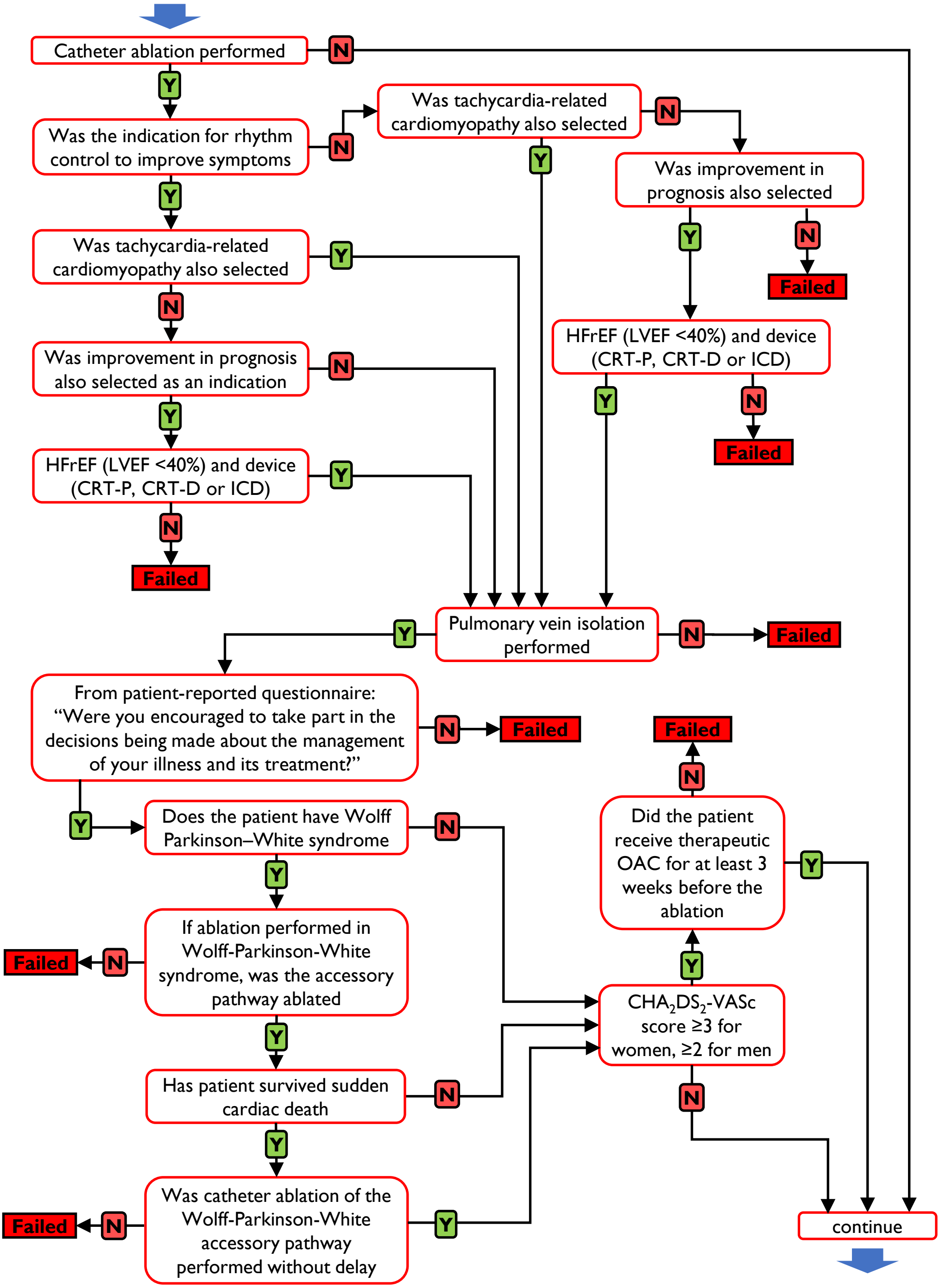
Catheter ablation performed
Was the indication for rhythm control to improve symptoms
Was tachycardia-related cardiomyopathy also selected
Was improvement in prognosis also selected
Was tachycardia-related cardiomyopathy also selected
Was improvement in prognosis also selected as an indication
HFrEF (LVEF <40%) and device (CRT-P, CRT-D or ICD)
HFrEF (LVEF <40%) and device (CRT-P, CRT-D or ICD)
Failed
Pulmonary vein isolation performed
From patient-reported questionnaire: "Were you encouraged to take part in the decisions being made about the management of your illness and its treatment?"
Does the patient have Wolff Parkinson–White syndrome
If ablation performed in Wolff-Parkinson-White syndrome, was the accessory pathway ablated
Has patient survived sudden cardiac death
Was catheter ablation of the Wolff-Parkinson-White accessory pathway performed without delay
Did the patient receive therapeutic OAC for at least 3 weeks before the ablation
CHA2DS2-VASc score ≥3 for women, ≥2 for men
continue
Y
N

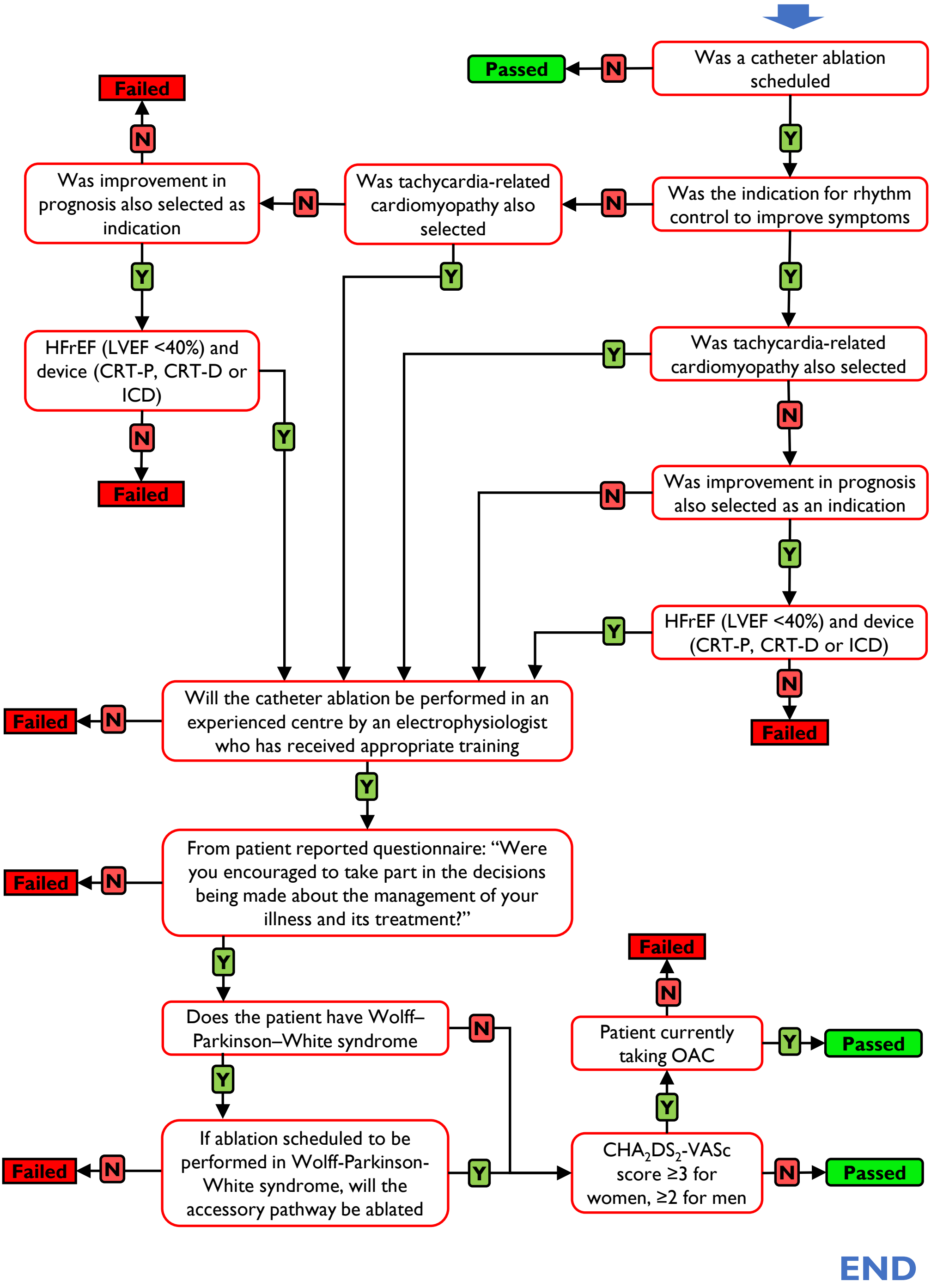
Was a catheter ablation scheduled
N
Passed
Y
Was the indication for rhythm control to improve symptoms
N
Was tachycardia-related cardiomyopathy also selected
N
Was improvement in prognosis also selected as indication
N
Failed
Y
HFrEF (LVEF <40%) and device (CRT-P, CRT-D or ICD)
N
Failed
Y
Y
Was tachycardia-related cardiomyopathy also selected
Y
N
Was improvement in prognosis also selected as an indication
N
Y
HFrEF (LVEF <40%) and device (CRT-P, CRT-D or ICD)
Y
N
Failed
Will the catheter ablation be performed in an experienced centre by an electrophysiologist who has received appropriate training
N
Failed
Y
From patient reported questionnaire: "Were you encouraged to take part in the decisions being made about the management of your illness and its treatment?"
N
Failed
Y
Does the patient have Wolff–Parkinson–White syndrome
N
Y
If ablation scheduled to be performed in Wolff-Parkinson-White syndrome, will the accessory pathway be ablated
N
Failed
Y
CHA2DS2-VASc score ≥3 for women, ≥2 for men
N
Passed
Y
Patient currently taking OAC
Y
Passed
N
Failed
END

ONLINE FIGURE S5: KEY MESSAGES OF THE STEEER-AF PROGRAMME

STEEER-AF

ESC European Society of Cardiology · EHRA European Heart Rhythm Association · ESC Council Stroke

Stroke prevention

Key actions for commitment to change

DO's	DON'Ts
**Prevention of stroke and thrombo-embolism**	
Do calculate and record the CHA_2DS_2-VASc score in all patients with AF	*Do not* use antiplatelet therapy for stroke prevention in patients with AF
Do offer oral anticoagulation to all patients with an elevated CHA_2DS_2-VASc score	*Do not* routinely offer anticoagulation to patients with a CHA_2DS_2-VASc score of 0
**Appropriate use of anticoagulation**	
Do give patients a direct/non Vitamin K oral anticoagulant for stroke prevention, instead of warfarin	*Do not* give a direct/non Vitamin K oral anticoagulant to patients with moderate-severe mitral stenosis or a mechanical heart valve
Do keep INR levels between 2.0 and 3.0 for patients on warfarin, with time in range >70%	*Do not* give low dose anticoagulation, unless the patient meets reduced dose criteria (table below)
**Anticoagulation in specific cases**	
Do delay starting anticoagulation immediately after an acute stroke	*Do not* combine anticoagulation with aspirin or other antiplatelets, unless an acute vascular event

	Apixaban	Dabigatran	Edoxaban	Rivaroxaban
Dose	5mg twice daily	150mg twice daily	60mg once daily	20mg once daily
Only reduce dose if:	Two of the following:<br>1. Weight ≤60kg<br>2. Age ≥80 years<br>3. Creatinine ≥ 133umol/L (1.5mg/dL)	Any of the following:<br>1. Age ≥80 years<br>2. On verapamil<br>3. High bleeding risk	Any of the following:<br>1. Weight ≤60kg<br>2. CrCl ≤50mL/min<br>3. On dronedarone, other P-Gp inhibitors	CrCl ≤50mL/min
Reduced dose	2.5 mg twice daily	110mg twice daily	30mg once daily	15mg once daily

CrCl = Creatinine clearance

STEEER-AF

ESC European Society of Cardiology · EHRA European Heart Rhythm Association · ESC Council Stroke

Rhythm control

Key actions for commitment to change

DO's	DON'Ts
**General management**	
Do assess and record symptoms using the modified EHRA score in all patients with AF	***Do not* forget** to assess and manage blood pressure, lifestyle risk factors and co-morbidities
Do make sure that patients take part in decisions about their AF management and treatment	*Do not* offer rhythm control for prognosis without symptoms, except for tachycardiomyopathy or HFrEF with implanted CRT/ICD
**Cardioversion**	
Do perform electrical cardioversion as soon as possible if there is haemodynamic instability in the patient	*Do not* perform pharmacological cardioversion in patients with AV node dysfunction or prolonged QT interval (>0.5s) without a pacemaker in-situ
Do take account of the following when thinking about pharmacological cardioversion: Co-morbidities; Cardiovascular risk; Risk of serious pro-arrhythmia; Extra-cardiac toxic effects; Patient preferences; Symptom burden	*Do not* use anti-arrhythmic drugs other than amiodarone for pharmacological cardioversion in patients with moderate or severe valve disease, HFrEF or coronary artery disease
Do ensure at least 3 weeks of effective anticoagulation prior to any cardioversion	*Do not* forget that transoesophageal echo can exclude thrombus before urgent cardioversion
**Long-term anti-arrhythmic drugs**	
Do consider adding anti-arrhythmic drugs to rate control therapy where symptoms could be improved by a rhythm control strategy	*Do not* prescribe anti-arrhythmic drugs to patients with permanent AF
After considering benefit vs risk, dronedarone can be used in patients with structural/valvular heart disease, and amiodarone in those with HFrEF	*Do not* offer flecainide, sotalol or propafenone to patients with coronary disease, heart failure, structural or moderate/severe valvular disease
**Catheter ablation**	
Do isolate pulmonary veins during catheter ablation for symptomatic AF	*Do not* perform ablation for AF unless the patient has at least 3 weeks of effective anticoagulation
Only attempt catheter ablation for AF in experienced centres with a trained operator	

HFrEF = heart failure with reduced ejection fraction
CRT = cardiac resynchronisation therapy
ICD = implanted cardiac defibrillator
